# A Review on Engineering Design for Enhancing Interfacial Contact in Solid-State Lithium–Sulfur Batteries

**DOI:** 10.1007/s40820-023-01306-z

**Published:** 2024-01-04

**Authors:** Bingxin Qi, Xinyue Hong, Ying Jiang, Jing Shi, Mingrui Zhang, Wen Yan, Chao Lai

**Affiliations:** School of Chemistry and Materials Science, Jiangsu Normal University, Xuzhou, 221116 Jiangsu People’s Republic of China

**Keywords:** Solid-state lithium–sulfur batteries, Solid-state electrolytes, Electrode/electrolyte interface, Interfacial engineering, Enhancing interfacial contact

## Abstract

The utilization of solid-state electrolytes (SSEs) presents a promising solution to the issues of safety concern and shuttle effect in Li–S batteries, which has garnered significant interest recently. However, the high interfacial impedances existing between the SSEs and the electrodes (both lithium anodes and sulfur cathodes) hinder the charge transfer and intensify the uneven deposition of lithium, which ultimately result in insufficient capacity utilization and poor cycling stability. Hence, the reduction of interfacial resistance between SSEs and electrodes is of paramount importance in the pursuit of efficacious solid-state batteries. In this review, we focus on the experimental strategies employed to enhance the interfacial contact between SSEs and electrodes, and summarize recent progresses of their applications in solid-state Li–S batteries. Moreover, the challenges and perspectives of rational interfacial design in practical solid-state Li–S batteries are outlined as well. We expect that this review will provide new insights into the further technique development and practical applications of solid-state lithium batteries.

## Highlights

- The engineering design principles for enhancing interfacial contact between the electrodes (Li anodes and S cathode) and solid-state electrolytes in solid-state Li–S batteries are classified and discussed.
- Research progresses of experimental strategies for reducing interfacial impedance in solid-state Li–S batteries are summarized.
- Challenges and future perspectives of rational interfacial strategies in solid-state Li–S batteries are highlighted.

## Graphical abstract

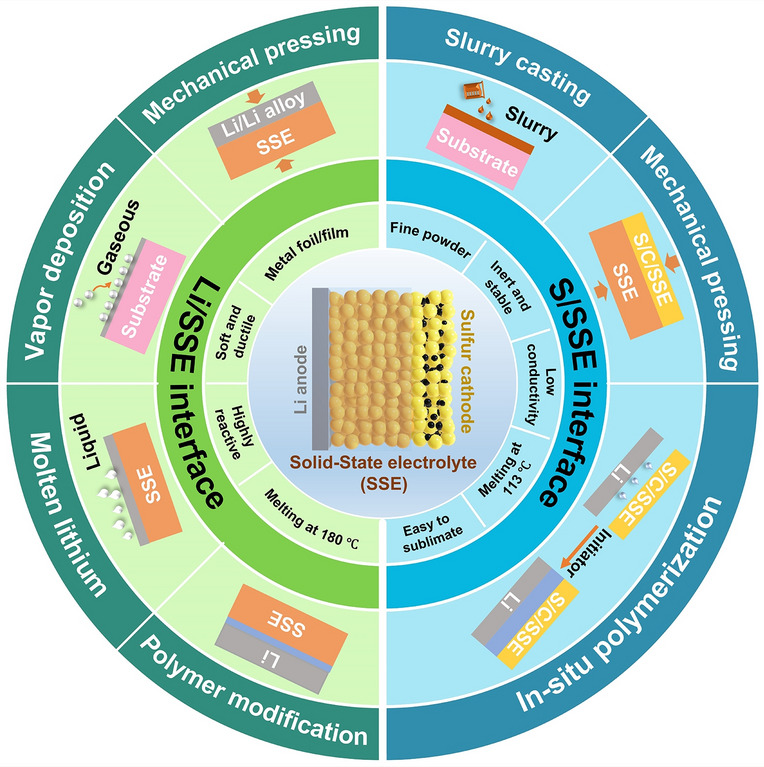

## Introduction

**Fig. 1:**
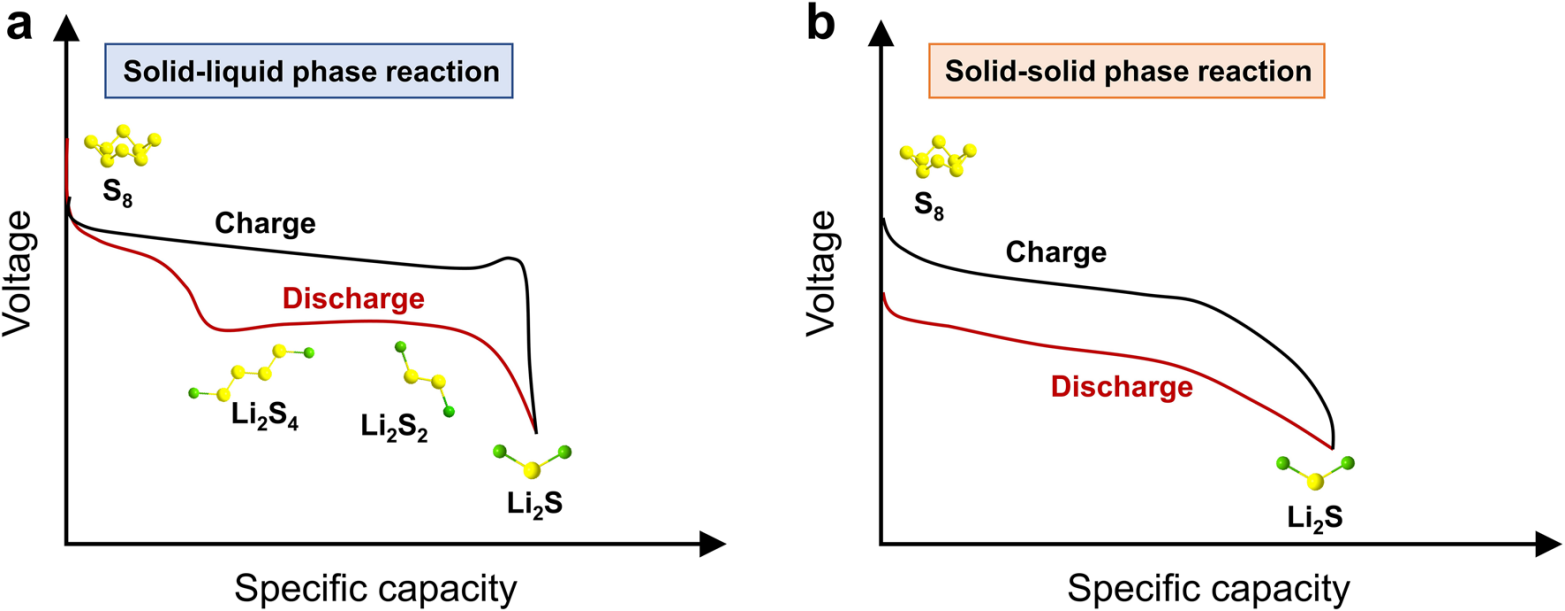
Typical charge/discharge voltage profiles of **a** solid–liquid phase reaction and **b** solid–solid phase reaction in Li–S batteries

As the energy crisis and environmental pollution issues worsen, the demand for renewable and highly efficient energy conversion and storage technologies is growing. Over the past decade, lithium-ion batteries (LIBs) based on the lithium-ion intercalation mechanism have undisputedly dominated the electrochemical energy storage market due to their high-energy density and long cycling stability [1]. The imperative task of producing rechargeable batteries with higher energy densities has become increasingly vital to meet the diverse needs of applications such as long-range electric vehicles, portable electronic devices, and smart grid storage. However, the state-of-the-art lithium-ion batteries encounter formidable obstacles in attaining an energy density that surpasses 400 Wh kg^−1^, primarily owing to the capacity constraint of the intercalation electrode materials [2]. Lithium-sulfur (Li–S) batteries, which are based on the redox conversion reactions of the metallic Li anode and the sulfur cathode, are considered as one of the most promising next-generation battery technologies, with the potential to attain high actual energy densities [3–6]. Specifically, the sulfur cathode possesses notable advantages, including high theoretical specific capacity of 1675 mAh g^−1^, cost-effectiveness, and environmental friendliness [7]. In terms of the anode material, it is worth noting that the lithium metal anode features an exceptional specific capacity of 3860 mAh g^−1^, alongside an impressively low equilibrium potential of − 3.04 V versus the standard hydrogen electrode. As such, it stands out as the most promising candidate for high-energy density battery systems [8]. Ever since the initial efforts in 1960s, a great deal of researches have been conducted with the aim of improving the energy density, prolonging the cycling lifespan, and avoiding the polysulfide shuttle in Li–S batteries [9–17]. In the typical ether-based electrolyte, the electrochemical reaction of sulfur is a complex multiphase and multistep process, involving the fracture and formation of S–S bonds, and the formation and conversion of a series of intermediate lithium polysulfides (LiPSs) [18–21]. The two-step process from sulfur to Li_2_S is characterized by the two plateaus in the discharge voltage profile. As illustrated in Fig. 1a, S_8_ is first reduced to LiPSs (Li_2_S_*x*,_ 4 ≤ *x* ≤ 8) at 2.3 V vs. Li^+^/Li, and further to Li_2_S at around 2.1 V versus Li^+^/Li, resulting in high theoretical capacity of 1675 mAh g^−1^. However, the commercialization of liquid electrolyte-based Li–S batteries continues to face many obstacles due to a range of underlying concerns and technological challenges [22–24]: (1) the insulating properties of elemental sulfur and discharged product Li_2_S and the sluggish redox kinetics; (2) the formation, dissolution, and shuttling of LiPSs intermediates in the liquid electrolyte; (3) the significant volume expansion of the sulfur cathode during lithiation; (4) the unstable interface between the Li anode and the liquid electrolyte, leading to capacity degradation and safety concerns. Various strategies have been proposed to mitigate these issues, including developing sulfur host materials which block the polysulfide diffusion and accelerate the conversion process, designing functional separators and interlayers, and optimizing electrolytes, etc*.*

One potential solution to address the challenges of the polysulfide shuttle in sulfur cathodes, as well as the safety hazard stemming from the unstable Li anode/electrolyte interface, is to replace liquid electrolytes with solid-state electrolytes (SSEs) [18, 22, 25, 26]. The use of SSEs, particularly inorganic SSEs, commonly leads to a solid–solid reaction route in Li–S batteries. The reaction process entails a direct transformation from elemental sulfur to Li_2_S without the formation of LiPSs. The solid–solid reaction of sulfur cathode exhibits a single discharge plateau at around 2.0 V versus Li^+^/Li (Fig. 1b). Therefore, utilizing SSEs is an effective approach to restrain the “shuttle effect”. In comparison to Li–S batteries that employ liquid electrolytes, solid-state Li–S batteries exhibit the following advantages [26]: (1) the improved cycling stability and increased energy efficiency resulting from the elimination of polysulfide formation and shuttling; (2) the significantly improved safety achieved by using nonflammable SSEs that do not evaporate upon thermal runaway, thereby mitigating the risk of hazardous fire accidents.

SSEs serve a crucial role in the functionality of solid-state batteries, acting as both a Li-ion conductor and a separator. In the view of ion conduction mechanisms, SSEs can be classified into two distinct categories [18, 22]: inorganic SSEs and polymer SSEs. Typically, inorganic SSEs exhibit high room-temperature ionic conductivity ranging from 10^−4^ to 10^−2^ S cm^−1^, high Li^+^ ion transference number close to 1, and excellent mechanical strength. Furthermore, it is noteworthy that inorganic SSEs can inherently circumvent the shuttle effect because soluble polysulfides are incapable of penetrating the SSEs. The inorganic SSEs that have been employed in lithium metal batteries (LMBs) include oxide SSEs (e.g., NASICON-type Li_1+*x*_Al_*x*_Ti_2−*x*_(PO_4_)_3_ (LATP), garnet-type Li_7_La_3_Zr_2_O_12_ (LLZO), and perovskites), sulfide SSEs (e.g., glass/glass–ceramic Li_2_S-P_2_S_5_, Li_10_GeP_2_S_12_, and Li_6_PS_5_Cl), nitride SSEs (e.g., Li_3_N and lithium phosphorus oxynitride (LiPON)), and hydride SEs (e.g., LiBH_4_) [27–29]. Among these inorganic SSEs, sulfide SSEs have garnered significant attention in solid-state Li–S batteries because sulfide SSEs are generally the most stable against the S_8_ molecule [30, 31]. In addition, sulfide SSEs possess extremely high room-temperature conductivities (10^−2^–10^−3^ S cm^−1^) and can be conveniently processed into anode/electrolyte/cathode tri-layered configuration through a cold-pressing technique without the requirement for a high-temperature sintering process. Recently, Kim et al*.* [32] synthesized a superionic halogen-rich Li-argyrodites using ultimate-energy mechanical alloying and rapid thermal annealing methods, in which they found Li_5.5_PS_4.5_Cl_1.5_ exhibited high Li-ion conductivity of 10.2 mS cm^−1^ at room temperature. Besides, lot of other sulfide SSEs, i.e., Li_10_GeP_2_S_12_ (12 mS cm^−1^) [33], Li_6.6_Si_0.0.4_S_5_I (14.8 mS cm^−1^) [34], Li_5.35_Ca_0.1_PS_4.5_Cl_1.55_ (10.2 mS cm^−1^) [35], Li_6.75_Sb_0.25_Si_0.75_S_5_I (13.1 mS cm^−1^) [36], were reported with a high ionic conductivity over 10 mS cm^−1^. Notwithstanding the promising attributes in solid-state Li–S batteries, the application of inorganic SSEs confronts severe difficulties that must be overcome. The poor wettability of inorganic SSEs against Li metal foil and their instability in ambient environments impede their applications in bulk-type LMBs. For example, most sulfide SSEs exhibit low chemical stability when exposed to moisture in air because sulfide materials tend to hydrolyze and generate H_2_S gas. Moreover, the high mechanical stiffness of inorganic SSEs can significantly increase the stress/strain at the electrode/electrolyte interface and is inadequate to accommodate the volumetric expansion during cycling, thereby leading to high interfacial resistance [37]. Introducing a buffer/protective layer between the electrodes and the electrolytes is one of the effective strategies to enhance the physical contact and to regulate the Li/SSE interface [31, 38–41]. For instance, the deposition of ZnO layer on the surface of garnet-like Ta-doped LLZO via atomic layer deposition significantly improved the wettability of the garnet SSE to Li anode, resulting in a conformal contact without interfacial void space [42]. Despite extensive researches have been conducted on this topic, poor interfacial capability and large thickness widely involve in the inorganic SSEs leads to the high resistance of batteries, which hinders their practical applications.

Alternatively, polymer SSEs composed of lithium salts (e.g., LiClO_4_, LiPF_6_, LiN(SO_2_CF_3_)_2_, etc*.*) dissolved in polymer matrices (e.g., polyethylene oxide (PEO), polyethylene glycol dimethyl ether (PEGDME), etc*.*), possess certain benefits over inorganic SSEs [43, 44]. First, polymer SSEs are highly flexible and elastic, which consequently enhances their interfacial compatibility, particularly on counteracting the volume variations that occur in the electrodes. Second, polymer SSEs exhibit the potential for scalable manufacturing by mature processes, such as solution casting, phase conversion, and electrostatic spinning. However, polymer SSEs exhibit extremely low ionic conductivity of 10^−8^–10^−6^ S cm^−1^ at room temperature. Therefore, in most cases, the batteries using polymer SSEs need to be operated at an elevated temperature above 60 °C. The deficient mechanical characteristics of polymer SSEs when subjected to high operating temperatures prove inadequate in suppressing Li dendrite growth. Developing novel polymer SSEs with high room-temperature ionic conductivity, high modulus and strength to resist the growth of Li dendrites is of significant importance for the fabrication of advanced Li–S batteries based on polymer SSEs [45–47]. Recently, Zhai et al*.* [48] designed 2D fluorinated graphene-reinforced PVDF-HFP-LiTFSI polymer electrolytes. The uniformly dispersed fluorinated graphene induced a unique grain refinement effect, which effectively improved the mechanical properties while maintaining high ion conductivity of 1.32 × 10^−4^ S cm^−1^ (30 °C). It should be noted that, similar to liquid electrolyte, the electrochemical process of sulfur cathode in polymer SSE-based Li–S batteries may also involve the generation and migration of polysulfide intermediates during cycling [49–51]. These LiPSs can still be dissolved in the polymer chain segments or plasticizers, and then migrate to the Li anode. The dissolved LiPSs passivate the Li anode, causing uneven lithium plating/stripping at the interface between the anode and the polymer SSEs. Overall, the challenges faced by solid-state Li–S batteries are considerably more severe than those in liquid electrolyte configurations, resulting in unsatisfactory cell performance. A critical issue is the manipulation of the interfaces in solid-state batteries.

**Fig. 2:**
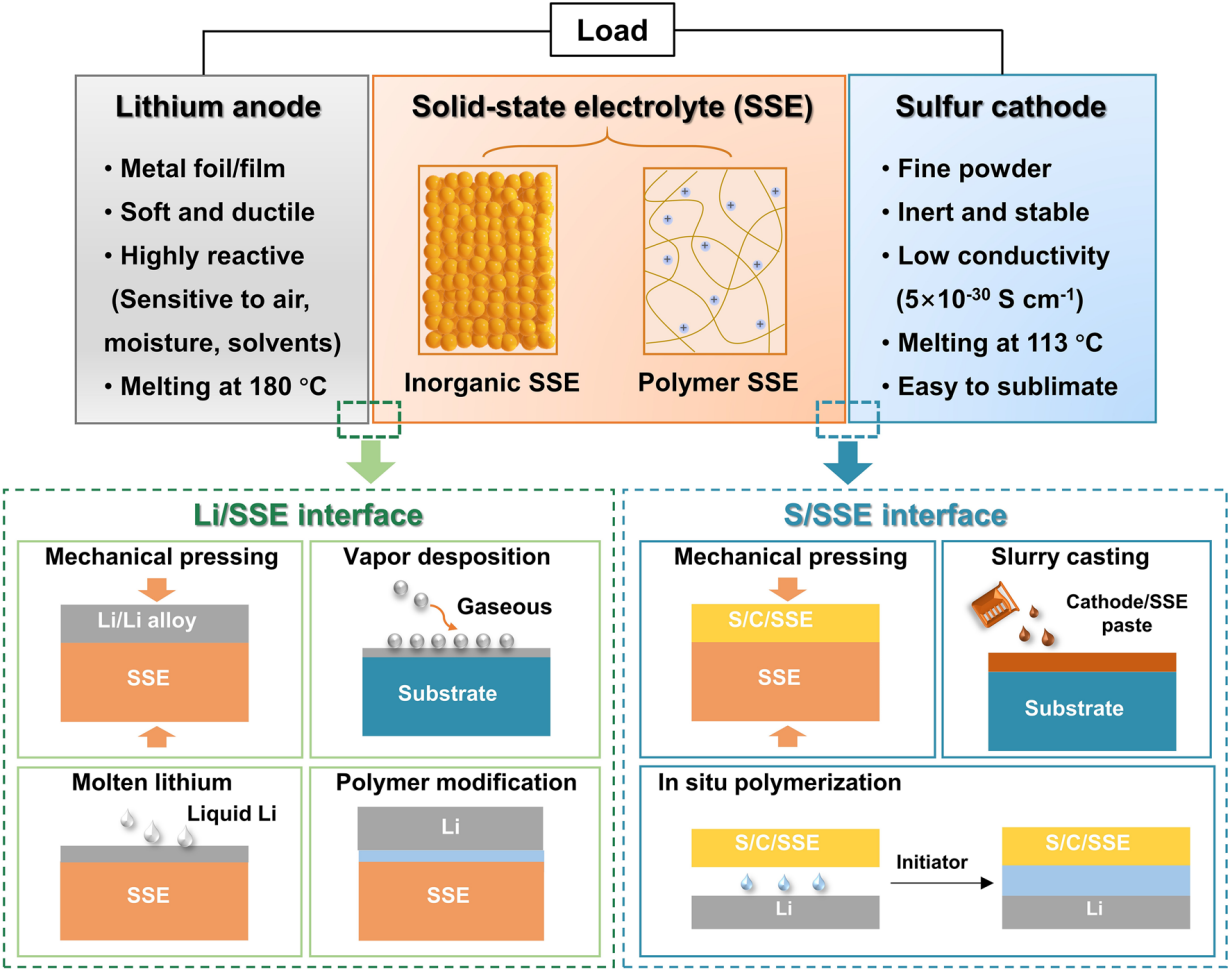
Schematic illustration of the experimental strategies for enhancing interfacial contact between the electrodes (lithium anodes and sulfur cathode) and solid-state electrolytes (SSEs) in solid-state lithium-sulfur batteries

The schematic diagram in Fig. 2 showcases a solid-state battery configuration consisting of lithium metal anode, SSE, and sulfur cathode, along with four types of interfaces: (I) Li/SSE interface; (II) the interface within the SSE (denoted as SSE/SSE interface); (III) S/SSE interface; and (IV) the interface within the sulfur composite cathode (denoted as S/S interface). Among these, the SSE/SSE interface impedance and the impedance within sulfur composite cathode are mainly caused by the limited contact area and the resistance at the grain boundary. For the electrode/electrolyte interface, it is important to consider not only the contact resistance, but also the differences in chemical and electrochemical potentials, which drive the elements to diffuse into each other and form interfacial phases that are detrimental to ion transport. In this review, the interface between the electrode and the electrolyte is the primary topic. According to the research of Nakamura et al*.* [52], the large electrode/electrolyte interfacial resistance of SSEs intensifies the uneven deposition of lithium and exacerbates the growth of lithium dendrite. This ultimately results in insufficient capacity utilization and poor cycling stability. Hence, the reduction of interfacial resistance between SSEs and electrodes is of paramount importance in the pursuit of high-performance solid-state LMBs [53–55]. When considering the electrolyte materials, it is acknowledged that inorganic SSEs, particularly those composed of oxides, exhibit more pronounced interfacial contact problems, whereas flexible polymer electrolytes adhere relatively tightly with electrodes, but also encounter some interfacial challenges during the dynamic process of battery cycling.

Owing to the unique redox mechanism of sulfur cathodes, the interfacial challenges encountered in solid-state Li–S batteries are not exactly the same as those of Li-ion batteries. The Li–S batteries employ lithium metal foil as the anode and cathode consisting of elemental sulfur or Li_2_S composite with carbon. The key to achieve optimal performance in solid-state Li–S batteries lies in the ability to achieve favorable interfacial compatibility and rapid charge transfer between materials possessing distinct chemical compositions. It is noteworthy that the physicochemical properties of the anode and cathode materials exhibit marked differences: lithium anode is highly active to many reagents, while sulfur-based cathode is chemically inert. Consequently, the methodologies employed in experiments to mitigate the interfacial impedance of anode/SSE and cathode/SSE exhibit dissimilarities. For example, sulfur can be dispersed in organic solvents to form a flowing paste and directly coat on the SSEs at room temperature via liquid casting method, but the Li anode foil can solely exist in a liquid state when heated to temperatures surpassing its melting point. Apart from the challenge of interfacial resistance between the electrodes and SSEs, there are different interfacial concerns associated with the lithium anode and the sulfur cathode, respectively. The primary obstacles of Li/SSE interface include the poor chemical durability of SSEs against Li and the uncontrolled dendrite growth during the cycling process. For sulfur cathode, sluggish charge transfer and polysulfide shuttle are critical interfacial issues. The variances in the electrochemical process have given rise to a multitude of distinct principles and experimental approaches to implement the interfacial strategies. In recent years, a number of high-quality review articles have summarized the evolution of SSEs and the electrode/electrolyte interfacial design in Li–S batteries [56–59]. Yu et al*.* [58] provided a survey of the research progress in the electrode/electrolyte interface in all-solid-state and hybrid electrolyte Li–S batteries. Umeshbabu et al*.* [59] symmetrically summarized the interfacial strategies in all-solid-state Li–S batteries based on the categorization of SSEs. Nevertheless, the strategies and experimental approaches used to achieve close contact at the Li/SSE interface and the S/SSE interface have been rarely discussed in solid-state Li–S batteries technically. In this review, we focus on the interfacial resistance issues and provide a comprehensive overview of the techniques employed in the integration of electrodes and electrolytes in solid-state Li–S batteries. The types of processing methodologies adopted to enhance interfacial contact can be broadly classified as follows (Fig. 2): mechanical pressing, vapor deposition, molten lithium, polymer modification, slurry casting, and in situ polymerization. We also summarize the electrochemical performance of solid-state Li–S batteries upgraded by the aforementioned strategies (Table 1) and discuss the features of these strategies. Finally, the challenges and prospects for the interfacial strategies are proposed, aiming to developing solid-state Li–S batteries.

**Table 1:** Summary of electrochemical performances of solid-state Li–S batteries employing various strategies to enhance interfacial contact

Anode	Cathode	Electrolyte	Interfacial strategy		Sulfur loading (mg cm^−2^) /Content	Ionic conductivity (S cm^−1^)	Current, working temperature	Initial discharge capacity (mAh g^−1^)	Capacity (mAh g^−1^)@nth cycle	Refs
			Anode	Cathode						
Li	L_i3_PS_4+5_/WVA-1500 carbon/PVC	β-Li_3_PS_4_	Pressing	Slurry casting	0.25–0.60	3.0 × 10^–5^ at 25 °C	0.1C, 60	1272	700@300	[60]
Li-In	Li_2_S-LiI/VGCF/75Li_2_S-25P_2_S_5_	75Li_2_S-25P_2_S_5_	Pressing	Pressing	0.9–1.1 (Li_2_S)	> 10^–4^ at 25 °C	2C, 25	980	980@2000	[61]
Li-In	S/CR	Li_10.05_Ge_1.05_P_1.95_S_12_	Pressing	Pressing	30 wt%	5.3 × 10^–3^	0.5C, N/A	~ 2100	1500@50	[62]
Li-In	S/C/Li_7_P_2.9_Sb_0.1_S_10.75_O_0.25_	Li_7_P_2.9_Sb_0.1_S_10.75_O_0.25_	Pressing	Pressing	0.73	1.61 × 10^–3^	0.05C, 60	1613	1365@100	[63]
Li-In	S/KB/Li_3_PS_4_-2LiBH_4_	Li_3_PS_4_-2LiBH_4_	Pressing	Pressing	2.57	6 × 10^–3^ at 25 °C	837.5 mA g^−1^, 60	1005	779@800	[64]
Li-Al	S/MWCNTs/Li_10_GeP_2_S_12_	Li_10_GeP_2_S_12_	Pressing	Pressing	1.07	N/A	0.2C, N/A	1237	1154@200	[65]
Li	S/AB/80Li_2_S-20P_2_S_5_	80Li_2_S-20P_2_S_5_	Vapor deposition	Pressing	35%	N/A	0.03C, 25	1350 (0.006 C)	900@20	[66]
Li-Al	S/C/PVP	Hybrid electrolytes (garnet and liquid)	Molten Lithium	Slurry casting	~ 1.0	N/A	N/A	1532	~ 1000@35	[67]
Li	S/C/binder	Hybrid electrolytes (Au coated garnet and liquid)	Molten Lithium	Slurry casting	0.9–1	7.5 × 10^–4^ at 25 °C	1C, 25	927	805@500	[68]
Li	S	Hybrid electrolytes (garnet and liquid)	Polymer modification	Melting diffusion	7.5	2.2 × 10^–4^ at 22 °C	0.2 mA cm^−2^, N/A	645	~ 550@30	[69]
Li	S/C/binder	LAGP	Polymer modification	Slurry casting	~ 1.0	5 × 10^–4^ at 20 °C	0.1C, N/A	1050	1080@150	[70]
Li-In	S-CuS/C/80Li_2_S-20P_2_S_5_	80Li_2_S-20P_2_S_5_	Pressing	Pressing	N/A	~ 10^–3^ at 25 °C	64 µA cm^−2^, 25	660	650@20	[71]
Li-Al	S/C/thio-LISICON	Thio-LISICON	Pressing	Pressing	25%	N/A	0.13 mA cm^−2^, 25	590	~ 420@5	[72]
Li-B	S/C/Li_7_P_3_S_11_	Ag@C modified LSPSCl	Pressing	Pressing	~ 1.50	4.1 × 10^–3^ at 25 °C	0.2C, 25	1316	1080@60	[73]
Li-In	S/C/Li_6_PS_5_Br	Li_6_PS_5_Br	Pressing	Pressing	20%	N/A	0.1C, 25	1355	1080@50	[74]
Li-In	Li_2_S/Li_6_PS_5_Cl/C	80Li_2_S-20P_2_S_5_	Pressing	Pressing	~ 3.6(Li_2_S)	1.3 × 10^–3^ at 25 °C	50 mA g^−1^, 25	648	830@60	[75]
Li	S-C/LiBH_4_	LiBH_4_	Pressing	Pressing	N/A	2 × 10^–3^ at above 117 °C	0.05C, 25	1140	~ 700@45	[76]
Li	rGO@S/AB/Li_10_GeP_2_S_12_	Li_10_GeP_2_S_12_ and 75%Li_2_S-24%P_2_S_5_-1%P_2_O_5_ bilayer	Pressing	Pressing	0.4–0.5	N/A	1C, 60	930	830@750	[77]
Li	Li_2_S-LiI/VGCF/LPS	Li_3_PS_4_-Kevlar	Pressing	Pressing	2.54(Li_2_S)	3 × 10^–4^ at 25 °C	0.2C, 25	537.8	~ 450@100	[78]
Li	S@LLZO@C	LLZO-PEO-LiClO_4_	N/A	Slurry casting	1.2	1.1 × 10^–4^ at 40 °C	0.05 mA cm^−2^, 37	1200	~ 900@200	[79]
CuF_2_ modified Li	rGO/S	PEO-LLZTO	N/A	Slurry casting	1.0	1.12 × 10^–3^ at 60 °C	0.5C, 60	875	647@250	[80]
Li	S/C	PEO-LiTFSI	N/A	Slurry casting	~ 0.6	N/A	0.2C, 60	967	768@100	[81]
Li	CMK-3/S	Poly-DOL electrolyte/separator	In situ polymerization		~ 2.0	> 1 × 10^–3^ at 25 °C	0.1C, 25	1140	~ 1000@5	[82]
Li	AC/S	Poly-DOL gel/separator	In situ polymerization		30%	N/A	1C, 25	683	454@400	[83]
Li	KB/S	Poly-DOL gel/separator	In situ polymerization		1.5	5.8 × 10^–3^ at 25 °C	0.5C, 25	1010	503@1000	[84]
Li	S	PETEA-based GPE/ separator	In situ polymerization		1.2–1.5	1.13 × 10^–2^ at 25 °C	0.5C, 25	650	530@400	[85]
Li	S@CMK-3	PETEA/PMMA based gel electrolyte	In situ polymerization		1.5–1.8	1.02 × 10^–3^ at 25 °C	3C, 25	625	574@500	[86]

## Strategies for Enhancing Interfacial Contact Between Li Anodes and SSEs

According to Monroe–Newman model for “dendrites” [87], SSEs with high mechanical modulus can effectively impede the growth of lithium dendrites. Actually, the phenomenon of lithium dendrite growth and lithium short circuits through SSEs remains prevalent during cycling of solid-state LMBs [88, 89]. The limited “solid–solid” interface contact between the Li metal foil and the rigid SSEs pellets hinders fast ion transport and engenders large interfacial resistance, ultimately resulting in the growth of lithium dendrites. The interface becomes even worse during the following plating/stripping process due to the generation of additional micro-voids and contact loss between Li anode and SSEs [90]. Therefore, it is imperative to ensure consecutive and conformal physical contact at Li anode/SSE interface during cycling for achieving uniform and stable plating behaviors.

Lithium foil anodes can be oxidized and deteriorated when exposed to the air and moisture environments. Meanwhile, lithium exhibits a high degree of reductivity, and it has the propensity to undergo chemical reactions with a multitude of chemical reagents. Therefore, the feasibility of the interfacial methods, including raw materials and ambient conditions, should be carefully considered when integrating SSEs with Li anodes. For example, treatment involving lithium anodes is usually carried out in an argon-filled glove box with O_2_ and H_2_O contents below 0.1 ppm. To improve the interfacial contact between SSEs and Li anodes, various experimental approaches, such as mechanical pressing, vapor deposition, molten lithium treatment, and polymer modification, have been widely employed during the assembly of solid-state Li–S batteries.

### Mechanical Pressing

Mechanical pressing is commonly used in the solid-state batteries using inorganic SSEs. Two types of external pressures are involved in the process. The external pressure during the preparation of electrodes and inorganic SSEs, as well as the cell assembly, can reach orders of several hundred MPa, or even several GPa, which is called the fabrication pressure. The external pressure applied during the time of cell operation is normally less than 100 MPa and is referred to as the operation stack pressure [91–93]. At present, most researchers fabricate the inorganic SSEs through a solvent-free process, by which one or more solid powders are mixed and then compressed into a dense and rigid body under external pressure [27, 93, 94]. The mechanical pressing is a commonly employed approach for enhancing the interfacial contact due to its practicality, scalability, and cost-effectiveness. To achieve a close physical contact between the inorganic SSE pellet and Li foil, the Li foil is pressed onto one side of the SSE pellet under a pressure of several hundred MPa or vice versa. In the research of Lin et al*.* [60], the Li_3_PS_4_ SSE/Li anode was pressed into a single block, in which 120 mg of *β*-Li_3_PS_4_ was directly cold-pressed on a 50 µm thick Li foil under external pressure of 240 MPa. Consequently, the Li–S battery delivered a good rate performance at 60 °C from 0.1 to 2C due to the compact structure and fast ion transport.

**Fig. 3:**
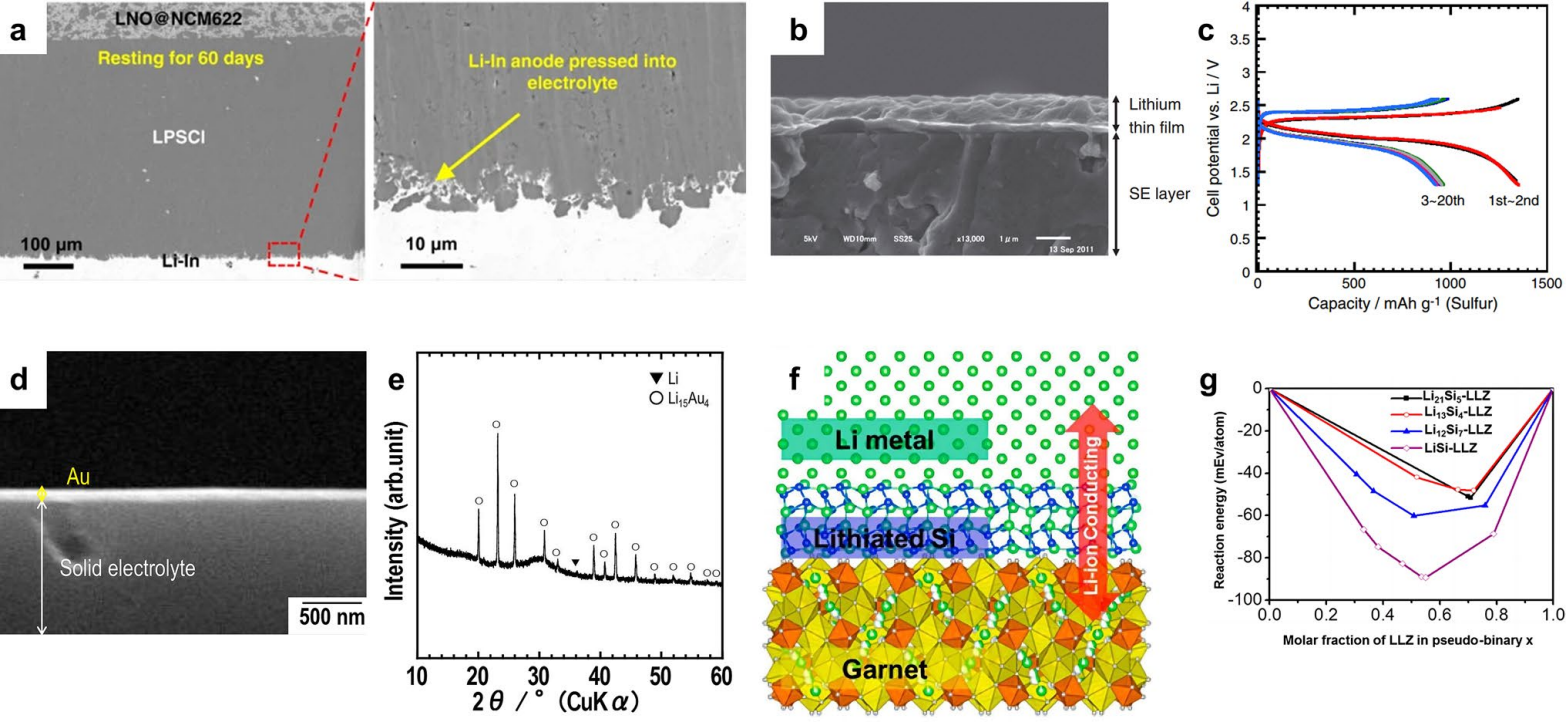
**a** Cross-sectional SEM images of Li-In|LPSCl|LNO@NCM622 cell with Li-In anode pressed onto the SSE after resting for 60 days without cycling. Reprinted from Ref. [97] with permission. Copyright 2021, Nature Portfolio. **b** Cross-sectional SEM image of a lithium thin film that was vacuum-evaporated onto SSE layer. **c** Charge–discharge curves of solid-state Li/lithium thin film/Li_2_S-P_2_S_5_ SSE/S cell at a temperature of 25 °C and current densities of 0.013 mA cm^−2^ and 0.064 mA cm^−2^. **b, c** Reprinted from Ref. [66] with permission. Copyright 2012, Elsevier B.V. **d** Cross-sectional SEM image of the interface between Au thin buffer film and Li_2_S-P_2_S_5_ SSE. **e** XRD patterns of Li/Au thin films on the Li_2_S-P_2_S_5_ SSE. **d**, **e** Reprinted from Ref. [98] with permission. Copyright 2016, Elsevier B.V. **f** Schematic representation of the “superlithiophilic” garnet-Li metal interface enabled by the lithiated Si layer formed in situ. Reprinted from Ref. [99] with permission. Copyright 2016, American Chemical Society. **g** Theoretical calculations demonstrate improved interfacial contact between LLZ and Li metal due to the enhanced wetting mediated by the lithiated Si interlayer. Reprinted from Ref. [99] with permission. Copyright 2016, American Chemical Society

Recent studies have reported a notable increase in SSE/Li interfacial resistance during cycling, which is attributed to the formation of unstable interphase due to the reactions between SSEs and the highly reductive lithium [90]. A routine strategy to inhibit such parasitic reaction is to introduce a thin buffer layer (such as indium [61–64], aluminum [65, 95], tin [96], silicon [96]) to avoid direct contact between the SSEs and Li anodes. These buffer layers, consisting of thin films or fine powders, can be sandwiched between the Li anode and the electrolyte, and subsequently fused at the interface under mechanical pressure. Indium metal with soft texture and good ductility is one of the most attractive choices for constructing buffer layers that form alloys with lithium and enable a stable electrolyte–electrode interface. In solid-state batteries, since the electrode and electrolyte are usually tightly fused and cannot be peeled off, the morphology analysis of the interface is usually based on the observation of the cross-sectional images. As evidenced by scanning electron microscope (SEM) image depicted in Fig. 3a, owing to the favorable deformability, Li-In alloy easily fills the pores and voids at the interface of the electrolyte layer when the cell is assembled under high pressure, which ensures uniform ion distribution and continuous charge transference [97]. Furthermore, it is generally acknowledged that Li-In alloys exhibit thermodynamic and kinetic stability toward SSEs. Hakari et al*.* [61] placed an indium foil with a thickness of 300 µm and a lithium foil with a thickness of 250 µm on the surface of the SSE as a counter-reference electrode, and then compressed them into an integrated block under 72 MPa. The indium buffer layer showed higher diffusivity of Li^+^ compared to pure lithium, which is advantageous for ion transport toward the interface; thus, uniform lithium plating can be achieved. In addition, the incorporation of other metals into lithium anodes may lead to a reduction in the lithium chemical potential, thereby mitigating the electrochemical decomposition of SSEs. As a result, the solid-state Li–S batteries utilizing 80Li_2_S·20LiI cathode, 75Li_2_S·25P_2_S_5_ SSE, and Li-In anode demonstrated a remarkable capacity exceeding 1100 mAh g^−1^ at 0.5C and retained a capacity of 980 mAh g^−1^ at 2C after 2000 cycles. Sakuma et al*.* [96] proposed that the Sn/Si powders with high ductility and particle size of approximately 50 µm could be pressed onto one side of the Li_3.25_Ge_0.25_P_0.75_S_4_ SSE under high pressure (184 MPa). Following this, a Li foil of 600 µm thickness was pressed onto the Sn/Si buffer layer at a pressure of 9.2 MPa to form Li-M (M = Sn, Si) alloy. The decrease in interfacial resistances was observed in the lithium symmetrical cells prepared in this way. The initial resistance was 160 Ω, which increased slightly in the subsequent cycles. By incorporating Li-M (M = Sn, Si) alloy via the simple pressing agglomeration approach, a stable solid-electrolyte interface (SEI) layer with high lithium diffusion rate and close contact conditions was achieved. The modification of the interface is confirmed to be an effective approach to reduce the interfacial resistance.

The strategy of applying external pressure has been universally adopted both in the fabrication step and cell operation process of solid-state batteries, with the aim of achieving intimate interface contact and inhibiting the formation of voids between different phases. Studies have demonstrated the effect of mechanical pressure on the electrochemical performance and correlated it with the interface stability and robust interparticle contacts [91, 100]. Although the application of external pressure is necessary for achieving optimal cell performance, the low yield strength of lithium may cause it to squeeze out through microcracks in the SSE, thereby leading to mechanically induced short circuits. The dendrite growth in grain boundaries of SSEs is even faster than in conventional liquid electrolytes, which prefers to penetrate through the grain boundaries and voids inside the electrolyte. Therefore, SSEs with compact structure are critical to prevent lithium dendrite formation. In addition, as cycling progresses, the accompanying volume changes of the lithium compounds and resulting internal stress during successive charge/discharge cannot be ignored, which induces the growth of lithium dendrites enclosing electrolyte particles, eventually leading to the failure of the contact between the anode and the electrolyte interface [101]. Therefore, a comprehensive consideration of the interfacial reaction, the mechanical properties of the electrode, and the interfacial changes during long-term cycling is necessary for tailoring the external pressure on the anode side of the solid-state LMBs.

### Vapor Deposition

The vapor deposition method refers to the technology of depositing materials in a gaseous or vaporized state onto the surface of a substrate. Under vapor deposition treatment, uniform, stable, high-purity solid sediment films or coatings can be attained with well-controlled thickness ranging from a few nanometers to several hundred micrometers depending on the procedure time. The utilization of vapor deposition facilitates the straightforward construction of compact and homogeneous films that are processed as thin-film Li anodes, LiPON-based SSEs or interfacial layers for solid-state LMBs [102, 103]. The process of vapor deposition can be classified into two main categories: physical vapor deposition (PVD) and chemical vapor deposition (CVD). PVD is a convenient and scalable deposition technique, whereby the source material is converted into a gaseous phase under vacuum condition. Subsequently, the resulting vapor is deposited onto the substrate surface to achieve high-purity and functional deposition film. In the realm of solid-state battery research, the dominant PVD processes include sputtering, pulsed laser deposition (PLD) and evaporation (thermal and electron beam) techniques.

It is noteworthy that PVD techniques do not enable the direct deposition of SSEs, but can be used to deposit thin-film Li anodes onto SSEs and to perform interfacial modification of SSEs. Processing of SSEs by PVD methods has been reported in the cases of LiPON, Li-rich garnets, NaSICON, sulfide SSEs, and perovskites [104]. Depositing ultrathin intermediary layer on SSEs to form the continuous interfaces by reacting with Li metal can increase the Li wettability of the SSEs surface and reduce the interfacial resistance. In 2012, Nagao et al*.* [66] reported the deposition of Li thin film with a thickness of about 1 µm onto the surface of Li_2_S-P_2_S_5_ SSE through the process of vacuum evaporation. First, the sulfur composite electrode and the Li_2_S-P_2_S SSE were uniaxially pressed into pellet to obtain favorable SSE/S contact. Then, thin lithium film was vacuum-evaporated onto the opposite side of Li_2_S-P_2_S_5_ SSE. Subsequently, the Li anode foil was attached onto the deposited Li film (Fig. 3b). The establishment of a close connection between the Li metal anode and Li_2_S-P_2_S_5_ SSE resulted in a decrease in interfacial resistance, bringing about the uniform deposition of lithium through the interface. This yielded a remarkable reversible capacity of 920 mAh g^−1^ after 20 cycles, as depicted in Fig. 3c. Unfortunately, the cell performance of this solid-state Li–S battery was investigated only at a low current density of 0.064 mA cm^−2^ (0.03C with respect to sulfur cathode). To improve the rate performance of solid-state batteries, a vacuum-evaporated indium metal film (~ 500 nm thick) was introduced between the Li anode and the Li_2_S-P_2_S_5_ SSE, which formed an alloy with lithium [105]. The utilization of an indium buffer layer showed higher lithium diffusivity in comparison to that of pure lithium. It has been verified that the insertion of this indium thin film at the interface between Li anode and SSE layer does not result in any alteration of the operating potential of the batteries. The control experiments validated that the cells prepared by evaporating indium on the SSE layer exhibited a higher capacity and a lower overpotential than the cells using indium evaporated on the Li foil. These findings suggested that the indium buffer film could efficaciously wet the SSEs interface and meliorate Li-ion transfer kinetics, thereby boosting rate performance at 0.13 mA cm^−2^. Similarly, Kato et al*.* [98] realized the interfacial integration of Li anode and Li_2_S-P_2_S_5_ SSE by modifying a 60 nm thick Au film at the electrolyte/anode interface under vacuum thermal evaporation (Fig. 3d). Afterward, ultrathin Li anode film with a thickness of about 3 µm was evaporated onto the Au film. X-ray diffraction (XRD) patterns of the Li/Au films vacuum-evaporated on the solid electrolyte indicated the formation of Li_15_Au_4_ alloy (Fig. 3e). The Au buffer layer exhibited a good compatibility with sulfide SSEs and a high Li-ion diffusion coefficient, thus resulting in stable Li plating/stripping and high utilization of Li anode. PVD is a mature and environmentally benign technology in terms of operation process and equipment, and has successfully applied in some industrial processes. However, the preparation of high-quality coating films by means of PVD technology requires high cleanliness of the substrate. In addition, due to the weak binding force between the deposited film and the substrate, the vacuum evaporated interfacial layer shows low durability against impact/wear, and may be damaged during electrochemical processes [102].

The CVD process involves the chemical reactions of gaseous gases on a solid substrate to deposit metallic or compound sediment layers on the substrate. One of the features of CVD is that the kinetic energy of the deposited particles is generally lower than that in most PVD techniques. This characteristic serves to reduce surface and film degradation during the growth process. Through the utilization of CVD technique, diverse types of thin films composed of metallic, inorganic, and organic materials can be synthesized to enhance interfacial contact. Amorphous LiPON is presently the only SSEs that can be created using vapor deposition processes. In the research of Kim et al*.* [106], amorphous LiPON thin films were deposited by CVD technique using lithium dipivaloylmethane, triethyl phosphate and NH_3_ as precursor materials. The LiPON electrolyte exhibited ionic conductivity of 2.95 × 10^−7^ S cm^−1^ at room temperature. Although the ionic conductivity is low, the surface resistance of LiPON SSE can be controlled at thin thicknesses (< 2 μm), allowing it to be used in the microelectronics. Similar to PVD, the CVD method can also be used for the purpose of modifying the interfacial layer between the lithium anode and SSEs. Luo et al*.* [99] proposed an interfacial strategy by depositing an ultrathin layer of amorphous silicon (~ 10 nm) onto Li_7_La_2.75_Ca_0.25_Zr_1.75_Nb_0.25_O_12_ (LLZ) SSE through CVD treatment. The wettability of LLZ SSE has changed from “super-lithiophobicity” to “super-lithiophilicity” as a result of the reaction between lithium and silicon and the in situ formation of lithiated Si. The interfacial stability between the LLZ SSE and the in situ-formed, lithiated Si was examined through first-principles calculations (Fig. 3f). The most thermodynamically favorable phase equilibria as interphase layers and their reaction energies were determined by considering the interface as a pseudo-binary system of lithiated Si and LLZ. The interfacial reaction energies are in the range of − 90 and − 40 meV atom^−1^ (Fig. 3g), implying kinetic stability and reduced interfacial deterioration. The preparation of silicon coating by CVD is a well-developed technology in the semiconductor industry and thus is expected to be directly applied to the large-scale production of solid-state LMBs. Nevertheless, the concerns of pollution and poisoning of the substrates should not be overlooked when modifying the Li anode/SSEs interface using CVD method. In the battery assembly involving vapor deposition procedure, the interface fusion between sulfur cathode and SSE is typically completed first, using techniques like mechanical pressing or solution casting. And then, thin film is deposited on the opposite side of SSE to improve the interfacial contact between Li and SSE. Such sequence protects the deposition film from solvent pollution or high-pressure damage.

**Fig. 4:**
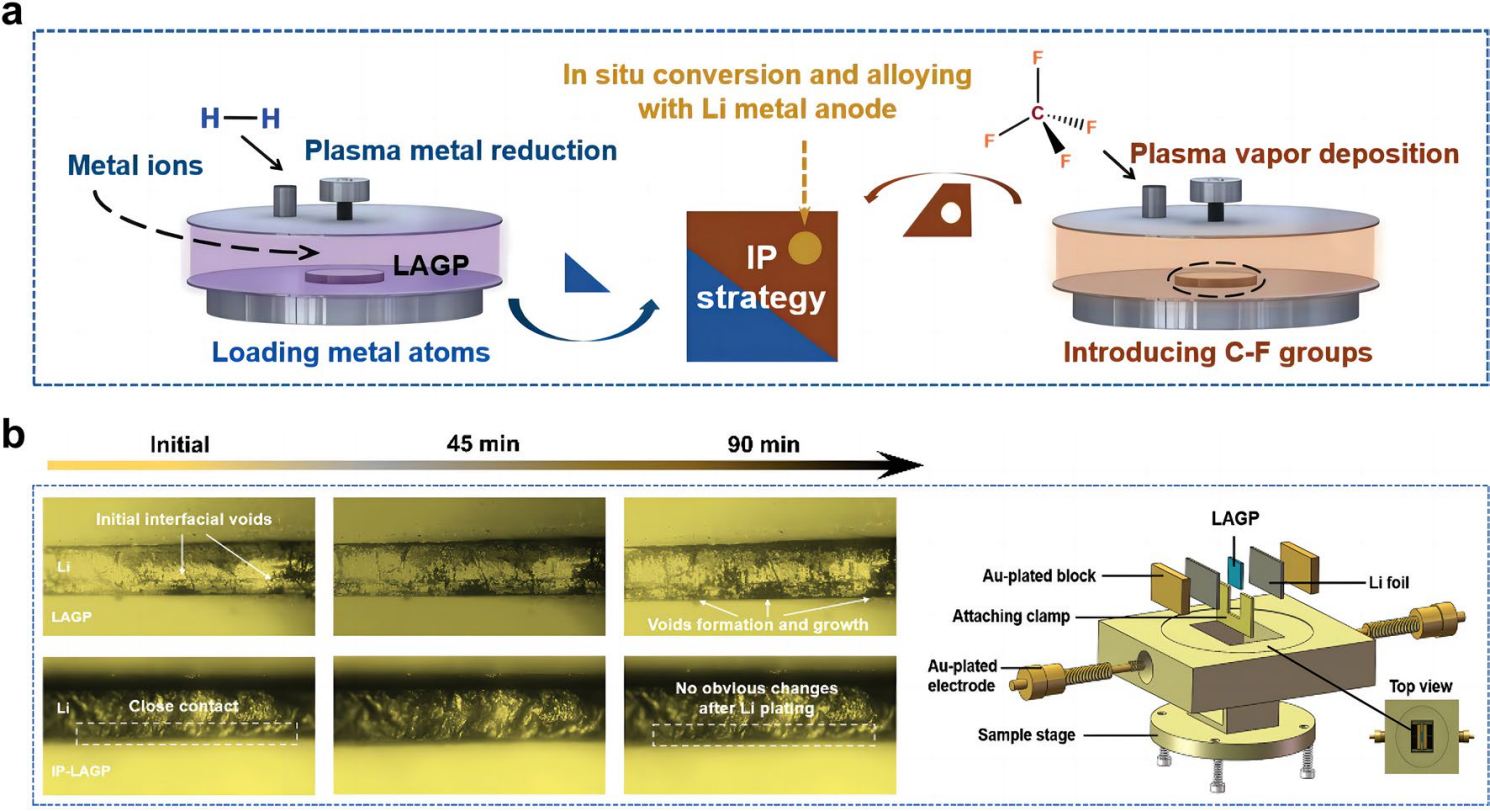
**a** Schematic of integration plasma (IP) strategy for constructing an interlayer layer between Li anode and LAGP SSE. **b** Optical microscope observation of the in situ evolution of LAGP/Li and IP-LAGP/Li interfaces during Li plating. Reprint from Ref. [38] with permission. Copyright 2023, Wiley–VCH GmbH

Sun et al*.* [38] reported an “integration plasma (IP)” strategy for constructing an interlayer layer between Li anode and SSE (Fig. 4a). First, Ag^+^-contained solution was sprayed over pristine NASCION-type Li_1.5_Al_0.5_Ge_1.5_P_3_O_12_ (LAGP) SSE pellets. Then, Ag^+^ was rapidly reduced to Ag atom and loaded onto the surface of LAGP under reductive agents. Next, CF_4_-contained gas drived plasma vapor deposition to produce C-F groups. Finally, the introduced Ag atoms and C-F groups in situ reacted with Li metal in the assembly process (preheated at 200 °C) and cycling of solid-state LMB to form composite interlayer containing Li-Ag alloy, crystalline LiF, and amorphous carbon. In situ optical observation was conducted to dynamically track the morphological evolution on the SSE/Li interface. As shown in Fig. 4b, the gaps and voids gradually extended during the Li deposition process, resulting in poor physical contact. In contrast, the IP-LAGP/Li interface exhibited close interfacial contact and high stability during cycling, owing to the synergy effects of the IP-formed interlayer to decrease surface roughness of LAGP and enhance chemical bonding between LAGP and Li anode. As a result, the composite interlayer enabled a fast Li^+^ diffusion and a high electron tunneling barrier on Li anode, thus delivering a high cycling stability for over 500 h at a high current density of 0.3 mA cm^−2^ with a capacity of 0.3 Ah cm^−2^.

### Molten Lithium Treatment

The lack of appropriate physical contact between Li anodes and SSEs results in high interfacial resistance (usually in the range of 100–1000 Ω cm^2^) and uneven current distribution. Consequently, the polarization of the cell becomes more intense as the cycle time and current density increase. To remedy the issues of high interface impedance, Sharafi et al*.* [107] preheated the battery to 175 °C and then cooled it to room temperature. The Li-LLZO interface resistance decreased dramatically from 5822 to 514 Ω cm^2^. Although the heating treatment did not reach the melting point of lithium (180 °C), this experiment demonstrated that the high-temperature treatment facilitated the diffusion of Li atoms, thus improving the interfacial contact between Li and LLZO.

**Fig. 5:**
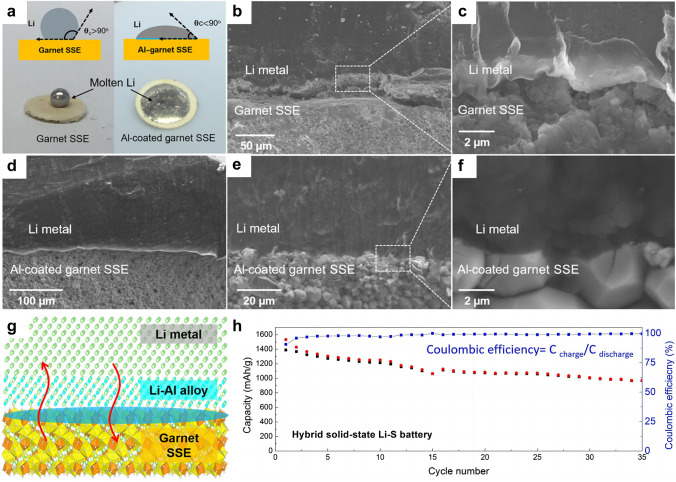
**a** Wetting behavior of molten lithium on the garnet SSE and Al-coated garnet SSE. **b, c** Cross-sectional SEM images of the interface between Li and garnet SSE, showing the poor Li wettability of uncoated garnet. **d-f** Cross-sectional SEM images of the interface between Li and Al-coated garnet SSE, exhibiting superior Li wettability. **g** Reaction between Al and Li promotes enhanced infusion of molten Li onto the garnet’s rough surface, whereas the formation of a Li-Al alloy fills the gap between the garnet solid electrolyte and the Li metal. **h** Electrochemical performance of the hybrid solid-state Li–S battery. Reprinted from Ref. [67] with permission. Copyright 2017, The American Association for the Advancement of Science

In order to achieve practical high-energy density LMBs, it is necessary to utilize thin lithium foils with an area capacity less than 4 mAh cm^−2^ to be paired with conventional cathodes (area capacity of 3–4 mAh cm^−2^), which requires Li anode thickness of approximately 20 µm. The production of thin Li anodes with a thickness of 15–30 μm through the conventional rolling of commercially thick Li foil poses a significant challenge due to the unfavorable mechanical property and sticky issue of metallic Li. Spreading molten Li onto current collectors or SSEs may be a promising strategy to realize the large-scale and cost-effective preparation of ultrathin Li anodes. Molten lithium exhibits the properties of high fluidity, remarkable surface smoothness, and high chemical activity. Utilizing a thermal infusion approach at a temperature of about 200 °C, the molten lithium is directly attached onto the dense SSE surface, achieving an ultrathin, SSE-supported lithium anode. However, the outcomes are not satisfactory since the problem of SSE wetting when exposed to molten lithium. It is well known that the poor wettability of molten lithium prevents it from spreading across the surface of the lithiophobic substrates [41, 108]. For example, in the case of pure garnet SSE, the molten lithium instantly forms a ball on the top of the garnet disk, indicating poor surface wetting behavior (Fig. 5a) [67]. Pretreating the surface of the SSEs through the modification of lithiophilic layer can considerably improve the feasibility of the molten lithium method. Fu et al*.* [67] fabricated an ultrathin, artificial intermediary layer by heating Li and Al foils together at 200 °C. The complete corrosion of Al foil indicated the diffusion of Al atoms into the molten Li. By forming this Li-rich solid solution, the SSE surface become lithiophilic, thereby allowing conformal adhesion of the bulk Li anode to the Li_7_La_2.75_Ca_0.25_Zr_1.75_Nb_0.25_O_12_ SSE surface upon solidification of molten Li. The interface morphology between the lithium anode and the electrolyte was characterized by SEM. As shown in Fig. 5b and 5c, the large gap between the garnet SSE (without Al coating) and lithium indicates that the uncoated SSE has poor wettability with lithium metal. In contrast, SSE with conformal Al coating shows intimate contact with lithium (Fig. 5d–f), and lithium fills the voids and grain boundaries, thus greatly increasing the interfacial contact area. The reaction between Al and Li promoted an increased infusion of molten Li onto the rough surface of SSEs (Fig. 5g). Additionally, the formation of Li-Al alloy served to fill the gap between the garnet SSE and the Li metal, thereby improving interfacial contact and enhancing the transport of Li^+^ ions. By forming an intermediary Li metal alloy, the interface resistance was reduced from 950 Ω cm^2^ (the pristine garnet/Li) to 75 Ω cm^2^ (the surface-engineered garnet/Li) at room temperature. The hybrid solid–liquid Li–S batteries were assembled and evaluated, where the solid electrolyte was on the anode side and the liquid electrolyte was applied to the cathode side. The hybrid solid–liquid Li–S batteries exhibited high capacities of ~ 1000 mAh g^−1^ and Coulombic efficiencies above 99% (Fig. 5h). These findings also suggest that the inorganic SSEs have the potential to effectively block the migration and shuttling of polysulfide in Li–S batteries. Similarly, Lu et al*.* [68] pre-coated Au thin layer on the surface of Li_6.4_La_3_Zr_1.4_Ta_0.6_O_12_ (LLZTO) ceramic, which functioned as a lithiophilic wetting interphase for the molten lithium. The enhanced interfacial contact was confirmed by electrochemical impedance spectroscopy (EIS) analysis of Li/LLZTO/Li symmetric cells, e.g., significantly reduced total resistance of the Li/Au-LLZTO-Au/Li (250 Ω cm^2^) than that of the Li/LLZTO/Li system (2500 Ω cm^2^).

### Polymer Modification

In contrast to the inorganic SSEs with rigid interface property, polymers typically inherit excellent flexibility and intimate interfacial contact with electrodes. A “softer contact” between polymers and Li anodes with larger contact area can wet the interfaces and depolarize the charge transfer process at the interfaces between electrolytes and anodes, which are conducive to reducing the interfacial resistance. Therefore, modifying the interface between Li anodes and inorganic SSEs using flexible polymer materials is one of the viable strategies to decrease interfacial resistance and improve cell cycling performance. Huang et al*.* [109] modified the LLZO SSE with conductive polydopamine coating. The modified surface showed lower interfacial resistance and higher ionic conductivity of 1.15 × 10^−4^ S cm^−1^ at 30 °C, which is nearly twice that of the unmodified SSE. Through the process of polymer modification, it is possible to augment the wettability and stability of interface between Li anodes and SSEs. In the research of Fu et al*.* [69], a PEO gel layer with a thickness of 2 µm was conformally coated on the garnet surface. The PEO interlayer ensured close contact between the garnet and lithium metal, and enabled homogeneous Li-ion flux through the interface. The results showed that the assembled hybrid Li–S cells exhibited remarkable Coulombic efficiencies over 99%, while accommodating a mass loading of up to 7 mg cm^−2^ for the sulfur cathode. Li et al*.* [70] modified the interface by spin coating the composite polymer slurry containing PEO, zeolite, and lithium bis(trifluoromethanesulphonyl)imide (LiTFSI) onto the LAGP. A thin, dense, and sticky polymer membrane adhered to the surface of the LAGP electrolyte. The polymer membrane exhibited a strong affinity toward the substrate, resulting in a notable enhancement of the interfacial wettability of the ceramic material. The solid-state Li–S battery based on the modified LAGP electrolyte exhibited high Coulombic efficiencies approaching 100%, and outstanding cycle stability with a capacity retention of 1080 mAh g^−1^ after 150 cycles at 0.1 C. Polymer modification of the interface is usually achieved by tape casting or spin coating the polymer solution followed by evaporation of the solvent, which can be readily scaled to fabricate low-cost solid-state LMBs.

## Strategies for Enhancing Interfacial Contact Between Sulfur Cathodes and SSEs

**Fig. 6:**
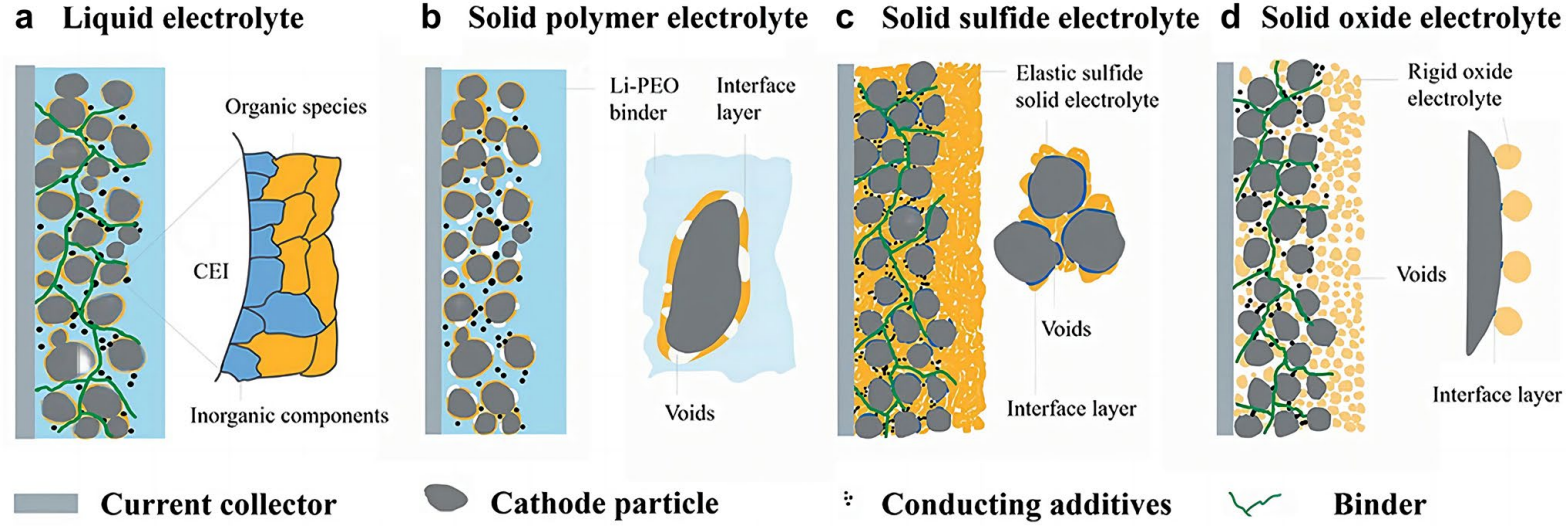
Interfacial models of the interfaces between the cathode and **a** liquid electrolyte, **b** polymer SSE,** c** sulfide SSE, and **d** oxide SSE. Reprinted from Ref. [110] with permission. Copyright 2018, Frontiers Media

The interfacial contacts between the cathodes and the electrolytes using different electrolyte systems have been explicated by the models depicted in Fig. 6a–d [110]. In liquid electrolyte system, the conventional method of producing sulfur cathode is to uniformly mix the powders of sulfur or Li_2_S, conductive agent, and polymer binder in an organic solvent. The as-obtained slurry is then coated onto the metallic current collector. It is noteworthy that the cathode particles can be uniformly wetted by the liquid electrolyte during the battery assembly, which consequently allows for the formation of ionically conductive cathode–electrolyte interphase (CEI) layer and the preservation of good contact between the electrode and liquid electrolyte throughout battery cycle (Fig. 6a). In solid-state Li–S batteries, the maintenance of Li-ion conduction pathway in the aforementioned case is a challenge as the SSEs cannot easily infiltrate into the cathode matrix. During cycling, the cathode experiences the redox conversion of sulfur, leading to alterations in both the morphology and elemental distribution of the sulfur cathode. Since the electrochemical reactions are contingent upon the contact between lithium ions, electrons, and active materials, interfacial design of sulfur cathodes/SSEs is essential. For polymer SSEs, which is elastic and flexible, the interfacial contact with cathodes is moderate. However, the formation of vacant cavities still reduces the effective contact area between sulfur cathodes and polymer SSEs due to the interfacial reaction and cathode pulverization during cycling (Fig. 6b). Among the inorganic SSEs, sulfide SSEs being ductile and deformable can maintain close contact with the cathode particles under mechanical pressing (Fig. 6c). The rigid ceramic nature of oxide SSEs causes the worst point-to-point contact with the sulfur cathode, which forms dead area caused by the isolated sulfur particles, ultimately resulting in large polarization and fast capacity fading (Fig. 6d). Due to the inherent rigidity of inorganic SSEs and sulfur cathodes, their physical contact is not dense enough. Moreover, the volume of sulfur cathode undergoes significant fluctuations during the electrochemical conversion from S_8_ to Li_2_S. As interface stress/strain intensifies, the interface contact between sulfur cathodes and SSEs deteriorates, resulting in rapid increase in battery impedance and decrease in utilization of active materials. Upon cycling, the aggravation of dead area due to the shrinkage and expansion of cathode particles, coupled with the growth of sulfur-rich needles at the cathode/electrolyte interface, expedite the deterioration of the battery. Therefore, the optimization of interfacial contact between sulfur cathodes and SSEs to establish unimpeded conductive pathway is essential for achieving superior electrochemical performance. Due to the distinguished properties of various types of electrolytes, there is distinct difference in the strategies adopted to enhance the interfacial contact between SSEs and sulfur cathodes.

### Mechanical Pressing

In order to reduce the interface impedance and establish smooth Li-ion transport pathway, ion-conductive SSEs powders are often blended with the sulfur/carbon composites and conductive agents during the preparation of sulfur cathodes for solid-state batteries, then followed by the application of external pressure pressing treatment in the battery assembly to yield an integrated composite pellet [65, 71–74]. In the composite sulfur cathodes, the mass fraction of the SSE is typically as high as 50 wt%, leading to extremely low sulfur content, typically below 40 wt%. By dispersing sulfur into a conductive matrix containing the electrolyte and carbon networks, it is possible to optimize the interface in the sulfur cathodes. For example, the cathode composite was initially formed by mixing S/CuS, Li_2_S-P_2_S_5_ glass–ceramic, and acetylene black carbon powders in a weight ratio of 20:30:3 [71]. Subsequently, the above cathode powder (10 mg) and the glass–ceramic electrolyte powder (80 mg) were placed within a polycarbonate tube and subjected to a pressure of 370 MPa. Following this, a Li-In foil as an anode was pressed onto the pellet under 120 MPa during battery assembly. The external pressure applied during the mechanical pressing procedure varies depending on the different interfaces, which is attributed to the distinct mechanical properties of lithium anode (soft and ductile) and sulfur composite cathode (rigid). Therefore, the typical sequence for assembling the battery involves first pressing the sulfur composite into contact with the electrolyte, followed by pressing the lithium or lithium alloy on the other side of the electrolyte. The integrated configuration formed by mechanical pressing significantly enhanced the interface contact between the electrodes and the Li_2_S-P_2_S_5_ electrolyte. To achieve closer contact between the sulfur and electrolyte, mechanical ball milling was carried out to reduce their particle sizes. The fine powder is suitable for facilitating intimate contact between the electrolyte and the electrode under mechanical pressing. Consequently, the cell exhibited high ionic conductivity of ~ 10^−3^ S cm^−1^ and retained reversible capacities over 650 mAh g^−1^ for 20 cycles.

**Fig. 7:**
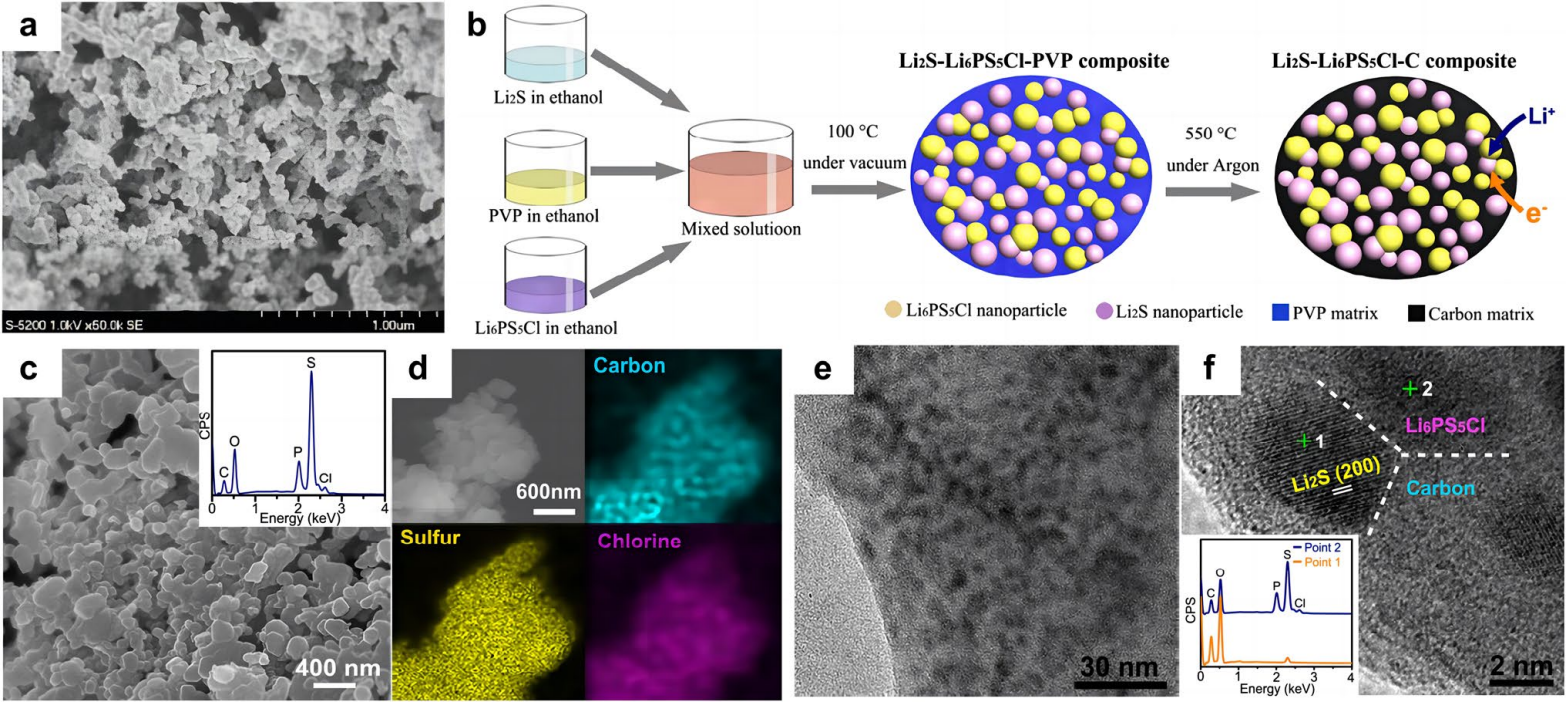
**a** SEM photograph of the sulfur composite prepared through high-temperature gas/solid mixing. Reprinted from Ref. [72] with permission. Copyright 2008, Elsevier B.V. **b** Schematic illustration of the bottom-up approach employed for the synthesis of the Li_2_S-Li_6_PS_5_Cl-C nanocomposite. **c** SEM image of the as-obtained Li_2_S-Li_6_PS_5_Cl-C nanocomposite. The inset shows the EDS result. **d** Elemental mappings of carbon, sulfur and chlorine in the composite. **e** TEM image of the Li_2_S-Li_6_PS_5_Cl-C nanocomposite.** f** High-resolution TEM image of the Li_2_S-Li_6_PS_5_Cl-C nanocomposite, and the inset shows the EDS results at point 1 and point 2, respectively. (**b-f**) Reprinted from Ref. [75] with permission. Copyright 2016, American Chemical Society

To achieve uniform distribution of the powder mixture, another common strategy employed prior to mechanical pressing is to heat the cathode composite at high temperatures, thereby inducing a transition of solid sulfur to either a liquid or gas state. Suzuki et al*.* [111] synthesized sulfur composite comprising elemental sulfur, acetylene black carbon, and Li_3.25_Ge_0.25_P_0.75_S_4_ electrolyte through high-temperature mechanical milling at 170 °C. The resulting composite was then pressed onto one side of the solid electrolyte for use in solid-state Li–S batteries. The process of high-temperature milling has been observed to promote the mobility of sulfur and decrease the particle sizes, thus yielding an improved specific capacity and enhanced cycle capability of the solid-state battery. Kobayashi et al*.* [72] prepared the nanocomposite of sulfur and acetylene black carbon through gas phase mixing at a high temperature of 300 °C. Following the amalgamation of gas and solid components, it was observed that the sulfur with large sizes from 1 to 10 µm were no longer present (Fig. 7a). Nanosized particles fabricated by the high-temperature gas phase mixing process play an important role in reducing the interfacial resistance. During the assembly of solid-state batteries, a cathode mixture which includes the sulfur nanocomposite, acetylene black carbon, and thio-LISICON (Li_3.25_Ge_0.25_P_0.75_S_4_) in a weight ratio of 1:1:2 was pressed onto one side of the electrolyte pellet at 500 MPa. High reversible capacity of ~ 900 mAh g^−1^ was obtained under the current densities of 0.013 mA cm^−2^. Upon increasing the current density to 0.13 mA cm^−2^, it was observed that the capacity remained 590 mAh g^−1^, implying that the issue of polarization is still a major obstacle in attaining optimal electrode utility at high current region.

The incorporation of the nanosized sulfur in close contact to either conductive carbon agents or electrolytes, followed by the uniform dispersion of these composites into an ionic/electronic conducting matrix, is expected to significantly enhance the electrochemical performances of solid-state Li–S batteries. Han et al*.* [75] reported a novel bottom-up method to synthesize a homogeneous nanocomposite by dispersing Li_2_S as the active material, polyvinylpyrrolidone (PVP) as the carbon precursor, and Li_6_PS_5_Cl as the SSE in ethanol (Fig. 7b). The subsequent steps included coprecipitation and high-temperature carbonization. The as-prepared Li_2_S-Li_6_PS_5_Cl-C composites showed irregular morphology with particle sizes ranging from 100 to 500 nm (Fig. 7c). More detailed information about the chemical composition and phase component can be obtained using energy-dispersive spectroscopy (EDS) and transmission electron microscopy (TEM). The EDS results confirmed the homogeneous distribution of carbon, oxygen, phosphorus, sulfur and chlorine in the composites (Fig. 7d). High-resolution TEM showed that Li_2_S and Li_6_PS_5_Cl, both possessing particle size of approximately 4 nm, were uniformly confined within the nanoscale carbon matrix (Fig. 7e, f). The utilization of homogeneous nanocomposite electrode, comprised of diverse nanoparticles possessing unique properties such as lithium storage capability, mechanical reinforcement, and ionic/electronic conductivity, has enabled the development of a mechanically robust and mixed conductive (ionic and electronic conductive) sulfur cathode for solid-state Li–S batteries. A large reversible capacity of 830 mAh g^−1^ (equivalent to 71% utilization of Li_2_S) was achieved at a current density of 50 mA g^−1^ after 60 cycles. Uniformly dispersing nanosulfur/carbon in a mixed conductive cathode can effectively reduce the volumetric fluctuations of sulfur throughout the charge/discharge process, thereby minimizing the associated mechanical stress/strain, and enhancing the cycle stability of solid-state Li–S batteries.

**Fig. 8:**
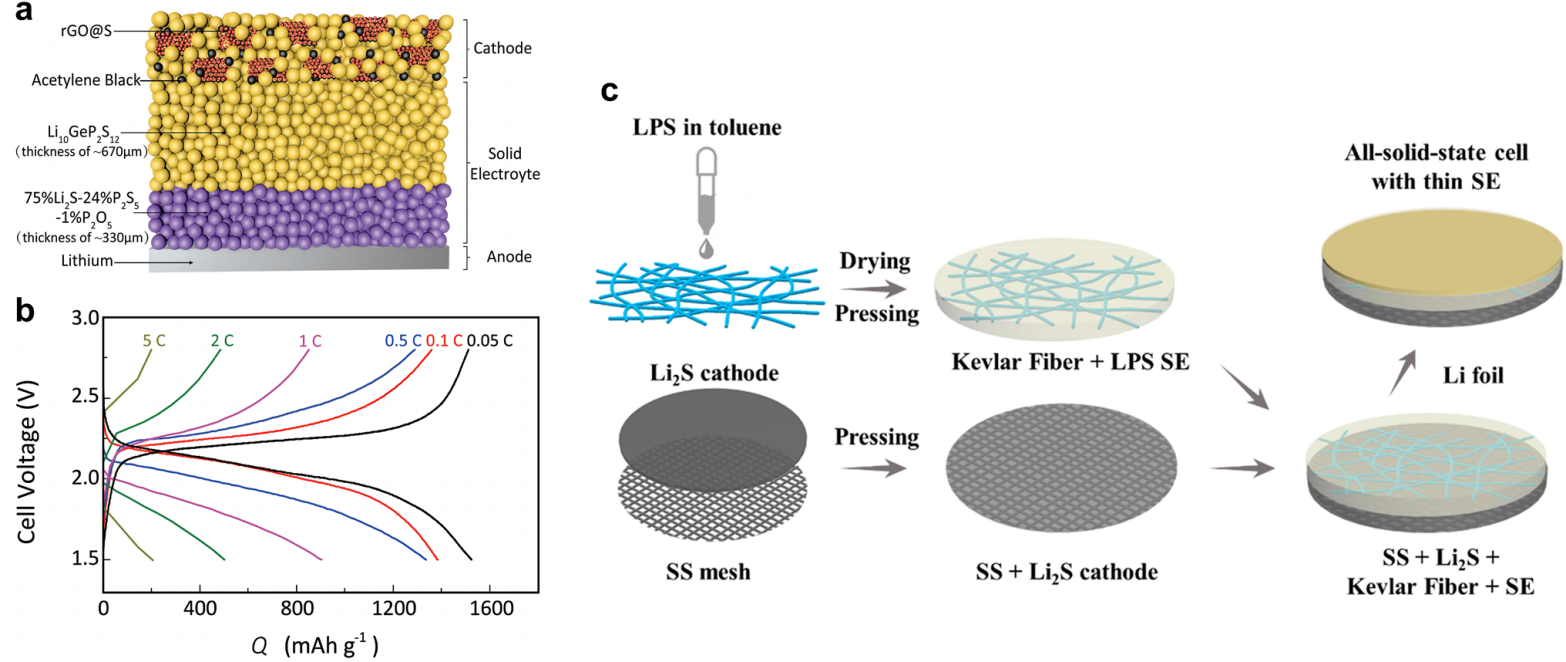
**a** Schematic diagram of solid-state Li–S battery composed of rGO@S-Li_10_GeP_2_S_12_-acetylene black composite cathode, bilayer SSE, and Li anode. Reprinted from Ref. [77] with permission. Copyright 2017, Wiley–VCH. **b** Galvanostatic charge/discharge profiles of the solid-state Li–S battery under different rates at 60 °C. Reprinted from Ref. [77] with permission. Copyright 2017, Wiley–VCH. **c** Schematic illustration of the manufacture process for the cathode-supported solid-state cell with a thin sulfide electrolyte. Reprinted from Ref. [78] with permission. Copyright 2019, American Chemical Society

The particle size of each component is one of the crucial parameters to achieve intimate interface contact in the sulfur/SSE composite. Other main factors, such as the properties of SSE powders, the incorporation of conductive agents, and the interfacial reaction that occurs between sulfur cathodes and SSEs, should also be taken into consideration. In the research of Unemoto et al*.* [76], a tight interface between the sulfur/carbon composite and the LiBH_4_ SSE powders was manifested by simple cold-pressing. This was due to the high deformability of the LiBH_4_ electrolyte. Yao et al*.* [77] proposed a solid-state Li–S cell consisting of rGO@S-Li_10_GeP_2_S_12_-acetylene black as composite cathode, bilayer SSE, and Li anode (Fig. 8a). The bilayer SSE is composed of Li_10_GeP_2_S_12_ and 75%Li_2_S-24%P_2_S_5_-1%P_2_O_5_. The implementation of Li_10_GeP_2_S_12_ with high ionic conductivity of 8.27 × 10^−3^ S cm^−1^ at room temperature in both the cathode and electrolyte layer is a strategic move to improve the ionic conductivity within the cell. The solid-state Li–S battery exhibited excellent rate performance of 1526, 1385, 1336, 903, 502, and 205 mAh g^−1^ at 0.05C, 0.1C, 0.5C, 1C, 2C, and 5C (Fig. 8b), respectively, which is comparable to that of Li–S battery in liquid electrolytes. In light of the instability of Li_10_GeP_2_S_12_ against lithium anode, a judicious intervention was undertaken by inserting a 75%Li_2_S-24%P_2_S_5_-1%P_2_O_5_ electrolyte layer that is more compatible with lithium. This measure was taken to preclude the occurrence of any deleterious reaction between the lithium anode and the Li_10_GeP_2_S_12_ electrolyte. The unique architecture endowed an intimate interface and uniform volume changes of sulfur, leading to an ultra-stable solid-state Li–S battery with good cycling stability (830 mAh g^−1^ at 1C for 750 cycles at 60 °C).

Conventional solid-state Li–S batteries start with the fabrication of the SSE layer, and then, the electrode layers are assembled on each side of the electrolyte under external pressure pressing. In this way, the SSEs must be sufficiently thick (> 200 µm) to withstand the high-pressure compression. Xu et al*.* [78] described a method for fabricating cathode-supported solid-state Li–S battery with a thin electrolyte (~ 100 µm). The Li_2_S composite cathode was cold-pressed alongside the supported stainless-steel mesh under 360 MPa (Fig. 8c). Subsequently, the Li_3_PS_4_ electrolyte suspension was immersed within Kevlar nonwoven scaffold, dried and cold-pressed onto the aforementioned sulfur cathode. The utilization of a stainless-steel mesh current collector effectively enhanced the mechanical integrity and interfacial stability of the Li_2_S cathode, which underwent huge volume change throughout the charge–discharge process. The Li-Li_2_S cell achieved high reversible discharge capacity of 949.9 mAh g^−1^ at 0.05C and stable cycling for 100 cycles at 0.2C.

Due to the high porosity of sulfur cathodes and electrolyte materials, post-assembly compression is generally regarded as an essential step in the fabrication process for achieving tight interfacial contact and electrochemical stability. It has been demonstrated that the external pressure applied during pressing has an effect on the microstructure and kinetic behavior of the composite cathodes. For example, the open-circuit voltage (OCV) of solid-state battery depends on the external pressure, showing a variation of approximately 1 mV/100 MPa [100]. However, excessive pressure can induce the rupture of active particles. The application of mechanical pressing facilitates the contact between the active particles and the electrolyte within the composite cathode, but after cycling, the volume fluctuation of the cathode particles causes gradual separation of the cathode/SSE interface.

### Slurry Casting

The utilization of slurry casting as a means of preparing sulfur cathode is widely recognized in liquid electrolyte systems. This process involves the mixing of sulfur-based powder, binder, and conductive agent in an organic solvent to yield a homogeneous slurry. Then, the slurry is cast onto the metallic current collector, i.e., the aluminum foil, and subsequently heat-dried to remove the solvent. In the solid-state Li–S batteries, it is feasible to achieve intimate interfacial contact by either directly casting sulfur-based slurry on the SSEs pellet or applying SSEs slurry on the sulfur cathode. For example, Lin et al*.* [60] prepared the sulfur cathode slurry by mixing Li_3_PS_4+5_ (60 wt%), WVA-1500 carbon (30 wt%), polyvinyl chloride binder in tetrahydrofuran solvent. Then, the cathode slurry was evenly cast on one side of the solid electrolyte pellet, followed by drying under vacuum at 80 °C. The uniform coating of the cathode slurry permitted the penetration of sulfur-based composite into the solid electrolyte, thereby enabling an intimate solid–solid contact between the cathode and the SSE.

**Fig. 9:**
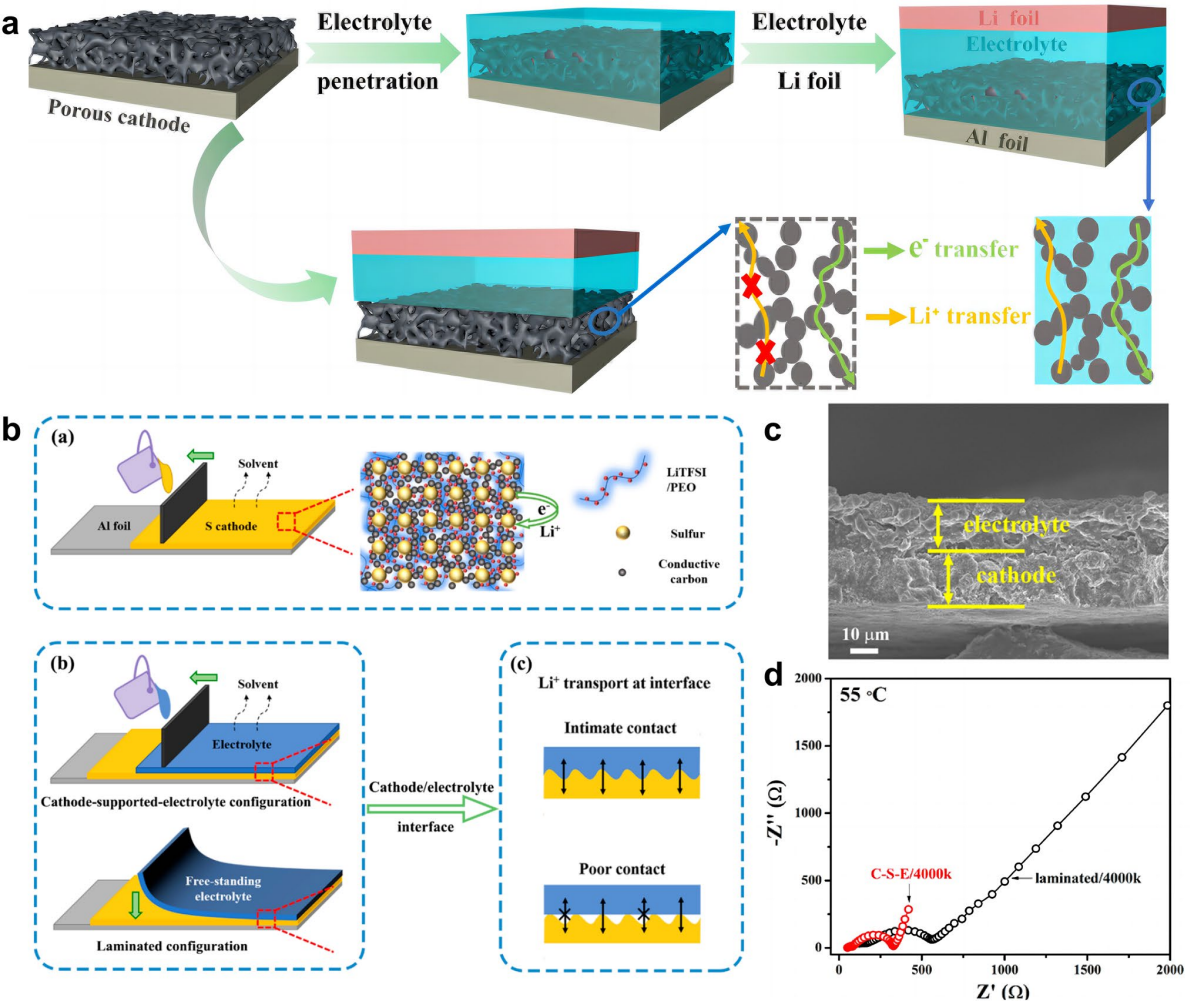
**a** Schematic illustration depicting the preparation of the electron/ion dual-conductive cathode framework and the battery assembly. Reprinted from Ref. [80] with permission. Copyright 2020, American Chemical Society. **b** Schematic illustration of the cathode-supported-electrolyte configuration through slurry casting, featuring interconnected electronic/ionic conductive networks. The comparison of fabrication procedures and Li^+^ transport for cathode-supported-electrolyte and laminated configurations.** c** Cross-sectional SEM image of the cathode-supported-electrolyte bilayer. **d** EIS comparison of batteries assembled by slurry casting and the conventional laminated polymer SSEs. **b–d** Reprinted from Ref. [81] with permission. Copyright 2020, American Chemical Society

In the second case of slurry casting, the electrolyte-based slurry can be directly coated onto the composite sulfur cathode. For example, the electrolyte-based slurry containing acetonitrile solvent, LLZO nanoparticles, PEO, and LiClO_4_ was cast onto the composite sulfur cathode [79]. The composition of the cathode and electrolyte were very close to each other, which helped to diminish the interfacial resistance that existed between the SSE and the cathode. In the research of Wang et al*.* [80], composite PEO-LLZTO/LiTFSI electrolyte was directly dropped on a highly porous sulfur/carbon cathode (Fig. 9a). The composite electrolyte penetrated into the porous structure of the sulfur cathode, thus forming a consecutive ionic/electronic dual-conductive framework. Following the integration of the electrolyte into the cathode, a notable reduction in battery resistance was observed, with a decrease from 6474 to 722 Ω. When utilized in Li–S batteries, it demonstrated high capacity of 925 mAh g^−1^ after 100 cycles at 0.1C and a capacity retention of 79.2%. In another study [81], a cathode-supported-electrolyte tape was successfully fabricated (Fig. 9b). PEO-based electrolyte slurry was cast over a composite sulfur/carbon electrode layer, which is totally different from the conventional approach of stacking the polymer membrane in a laminated configuration between electrodes. It can be observed that the cathode and electrolyte layers are intimately integrated, devoid of any voids or loose contact (Fig. 9c). The resulting solid-state Li–S battery exhibited reduced interfacial resistance in comparison to the ordinary laminated sample, as evidenced by the EIS plots (Fig. 9d).

The slurry casting improves the wetting capability of SSEs on sulfur cathodes and enhances the interface adhesion. Moreover, through the implementation of slurry casting, it is possible to decrease the thickness of the electrolyte to approximately 20 μm as opposed to the conventional SSEs that have a thickness exceeding 200 μm. This reduction in thickness facilitates faster reaction rates by minimizing ion diffusion path and improves the energy density of the battery. From a technical point of view, the slurry casting method is simple, easy-to-scale, and can be seamlessly jointed with the existing electrode preparation technology, which is very conducive to the industrialization of solid-state batteries. Notwithstanding, the issue of sluggish ion transport in solid–solid interface still exists, and the bonding strength between the coating and substrate is not strong, which renders the interface contact susceptible to morphology deterioration owing to the cathode’s volume change during the charge and discharge cycles. Moreover, certain SSEs, such as sulfides, exhibit a propensity to engage in chemical reaction with sulfur in the presence of a polar solution, thereby making the selection of an appropriate solvent for slurry preparation a challenging task.

### In Situ Polymerization

**Fig. 10:**
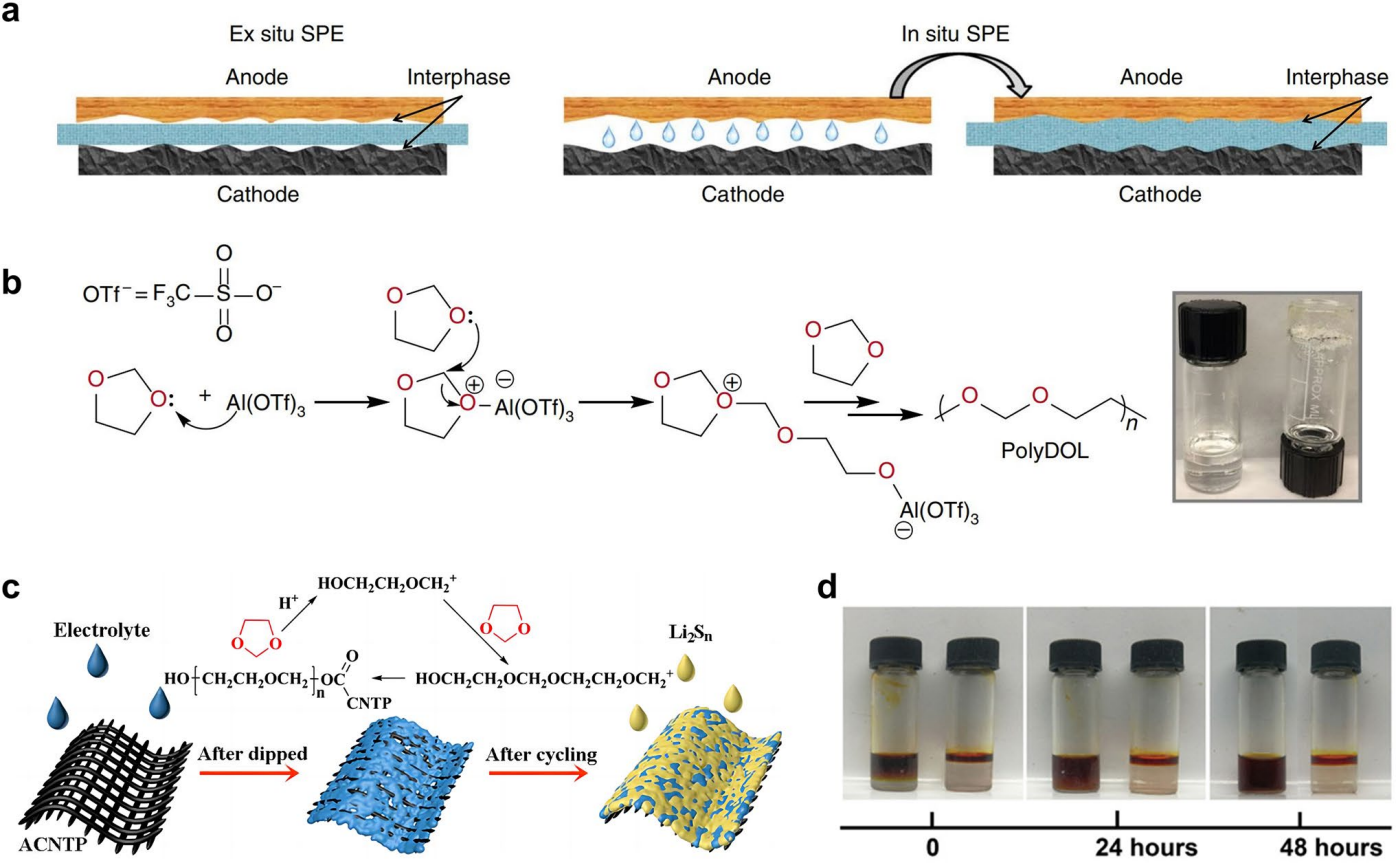
**a** Schematic diagrams that elucidate the ex situ and in situ synthesis of polymer SSEs. Reprinted from Ref. [82] with permission. Copyright 2019, Nature Portfolio. **b** Reaction mechanism illustrating how Al(OTf)_3_ initiates polymerization of DOL. Inset: digital photograph depicting the liquid DOL electrolyte (2 M LiTFSI/DOL, left) and solid-state poly-DOL electrolyte formed spontaneously in an electrolyte containing 0.5 mM Al(OTf)_3_ salt (right). Reprinted from Ref. [82] with permission. Copyright 2019, Nature Portfolio. **c** Illustrations of the polymerization of DOL induced by ACNTP in the electrolyte. Reprinted from Ref. [83] with permission. Copyright 2017, Royal Society of Chemistry. **d** Permeation behavior of Li_2_S_8_ in LE (left) and GPE (right). Reprinted from Ref. [84] with permission. Copyright 2018, The American Association for the Advancement of Science

The in situ formation of polymer SSEs through the polymerization of a liquid precursor is a promising approach to tackle the issues of low ionic conductivity and high interface resistance commonly observed in solid-state batteries. The in situ polymerization method can improve the interfacial contact of the SSEs with both the lithium anode and the sulfur cathode. In a broader context, the issue of interfacial contact in the sulfur cathode side is of significant concern, so we categorize this approach as part of the cathode interfacial domain. Generally, the in situ polymerization process entails the injection of liquid electrolyte into the battery during the battery assembly. As shown in Fig. 10a, the ex situ polymer SSEs deliver higher resistance as a result of poor contact with other cell components [82]. The liquid electrolyte precursors exhibit the capability to wet electrodes and create favorable interfacial contact with both the cathodic and anodic regions. This contact is preserved when the electrolyte is polymerized into a solid state. The precursor electrolyte usually contains monomer/oligomer molecules featuring unsaturated bonds (e.g., 1,3-dioxolane (DOL), pentaerythritol tetraacrylate (PETEA), ethoxylated trimethylopropane triacrylate (ETPTA), etc*.*) and lithium salt (e.g., LiTFSI, LiPF_6_, lithium difluoro(oxalato)borate (LiDFOB), etc*.*) [112]. Upon undergoing the in situ polymerization reaction induced by thermal, initiator-based, or electrochemical treatments, the liquid precursor electrolyte solidifies into an integrated solid/quasi-solid electrolyte that intimately interfaces with the battery. This process serves to enhance the compatibility of the solid–solid interface within the battery, ultimately leading to a marked improvement in the battery performance [113]. In addition, compared with the ex situ synthesis of SSEs, in situ polymerization simplifies and streamlines the battery assembly process [114].

As a widespread use low-molar-mass ether solvent, the ring-opening polymerization of DOL has been studied for over 50 years. Upon exposure to water, LiPF_6_, certain organo-aluminum compounds, such as diethyl aluminum chloride and ethyl aluminum dichloride, or under electrochemical processing, the DOL undergoes polymerization reaction to form poly-DOL [115–117]. In 2007, Kong et al*.* [116] proposed the in situ fabrication of lithium polymer battery based on the electro-polymerization of liquid electrolyte containing 1 M LiTFSI in DOL and 1,2-dimethoxyethane (DME). The broad anodic peak ranging from 3.8 to 4.3 V correlated to the polymerization process of DOL. Fourier transform infrared spectroscopy results and SEM characterization also confirmed the in situ formation of a smooth and uniform polymer electrolyte layer on the electrode. It is evidenced that although the electrochemical treatment under any current rate can induce the polymerization of DOL, the ionic conductivity of the resultant polymer substance is strongly influenced by the employed current rates. When the current rate was boosted to 500 mA g^−1^, the polymer electrolyte exhibited better cyclability comparable to those treated at lower current rates. This suggests that the properties of poly-DOL are influenced by the polymerization condition. In addition to the electrochemical treatment, it has been observed that Lewis acids can also prompt the polymerization of DOL. According to the study of Zhao et al*.* [82], the utilization of aluminum triflate (Al(OTf)_3_) as an initiator to induce the ring-opening polymerization of DOL within an electrochemical cell enabled the preparation of polymer SSEs with room-temperature ionic conductivity of mS cm-1 levels and low interfacial impedance (Fig. 10b). Li–S cells using this poly-DOL electrolyte displayed remarkable Coulombic efficiencies close to 100% and improved cycling performance as compared to the conventional liquid DOL electrolyte.

Xu et al*.* [83] found that the acidified carbon nanotube paper (ACNTP) interlayer could initiate the in situ polymerization of DOL solvent to form an ion-selective and self-healing solid electrolyte barrier in Li–S batteries (Fig. 10c). The flexible solid electrolyte membrane, measuring approximately 100 nm in thickness, achieved sufficient interfacial contact with the sulfur cathode. Furthermore, the resultant solid electrolyte membrane could seal the soluble polysulfides in the cathode region of the cell, while simultaneously permitting the bidirectional transport of Li^+^ ions. As a result, the assembled Li–S batteries showed good cyclic stability with specific discharge capacity of 454 mAh g^−1^ at 1C after 400 cycles, and high Coulombic efficiencies up to 99%. Liu et al*.* [84] proposed a method for upgrading conventional liquid electrolyte to GPE through the in situ ring-opening polymerization of DOL, which was initiated by LiPF_6_ in the presence of trace water. Because the GPE can still be regarded as an ether-based electrolyte with a unique quasi-solid existence form, it is the seamless alternative for conventional liquid electrolytes, which effectively limits the diffusion of polysulfide and the consequent “shuttle effect”. Such an in situ polymerization process drastically reduces the contact resistance between cathode materials and the electrolyte, thereby realizing the rapid transmission of Li^+^ ions. Significantly, the effect of polymer framework as blockage for polysulfide migration was demonstrated by the permeation test (Fig. 10d). After 24 h, the polysulfide still did not penetrate the GPE, reflecting its trapping behavior.

**Fig. 11:**
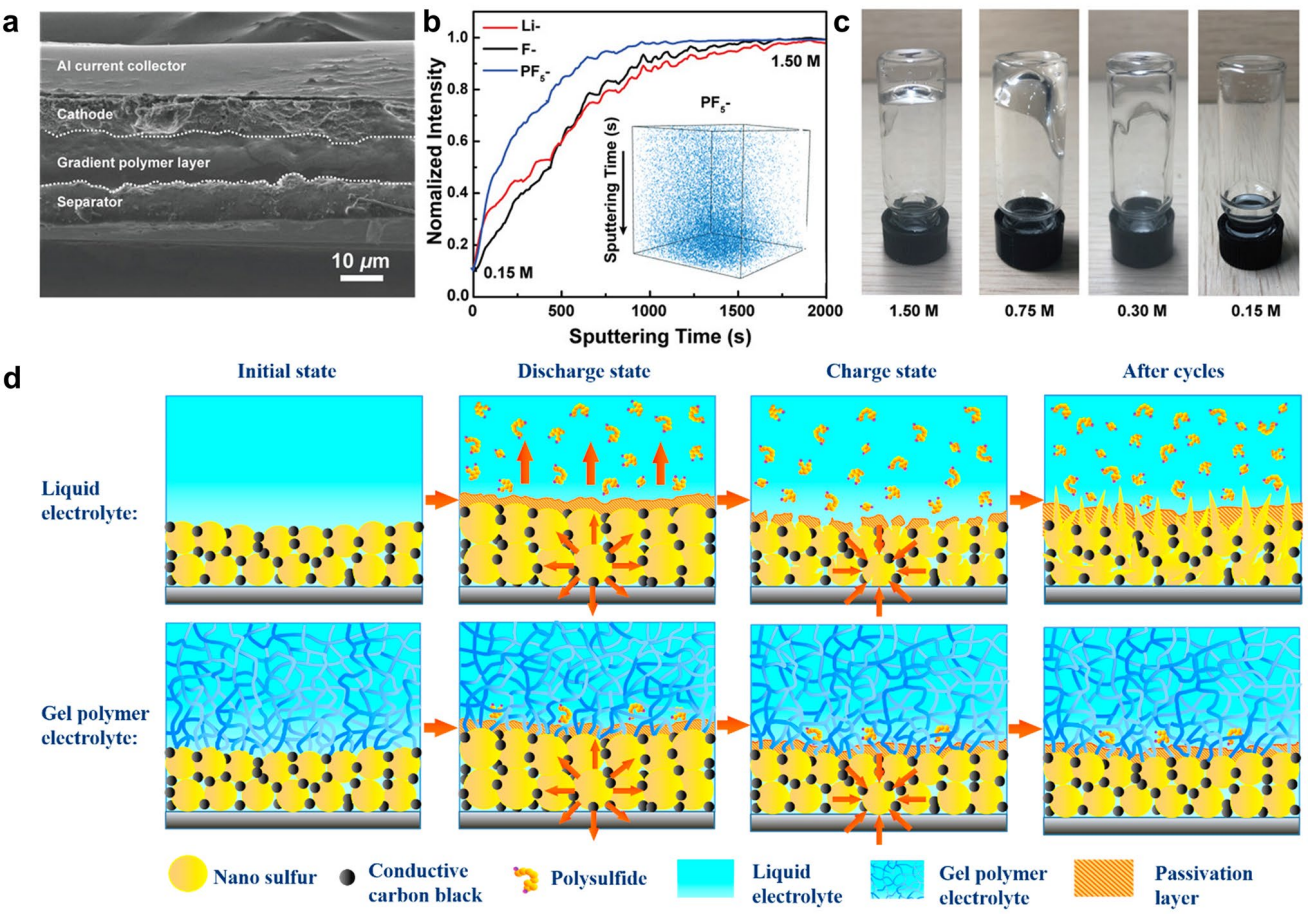
**a** Cross-sectional SEM image illustrating multilayered structure from the separator to the cathode. **b** ToF–SIMS depth profiles obtained from the in situ gel polymer interlayer, in which the inset shows the 3D spatial configuration of PF_5_^−^ signal. **c** Optical images showing the various polymerization degrees of liquid electrolyte with different LiPF_6_ concentrations. **a–c** Reprint from Ref. [118] with permission. Copyright 2020, WILEY–VCH Verlag GmbH & Co. KGaA, Weinheim. **d** Immobilization mechanism for polysulfides by capitalizing on PETEA-based GPE as electrolyte. Reprinted from Ref. [85] with permission. Copyright 2016, Elsevier Ltd

Wang et al*.* [118] developed an in situ interfacial polymerization (IsIP) strategy to form hybrid electrolyte between LiPF_6_-coated separator and sulfur cathode. LiPF_6_ initiated the in situ cationic polymerization of DOL, resulting in a gradient solidification on the cathode surface to form GPE. Meanwhile, the liquid electrolyte was retained within the cathode for rapid Li^+^ transport. A prototype Li–S cell was fabricated to inspect the in situ polymerization of ether electrolyte. According to the cross-sectional SEM image in Fig. 11a, a solid layer appears between the cathode and the separator after the IsIP process, indicating substantial formation of dense polymer electrolyte in close contact with the electrodes. To analyze the gradient polymerization process, time-of-flight secondary ion mass spectrometry (ToF–SIMS) was employed to depict the spatial distribution of chemical species alongside the cross section of the gel electrolyte. According to the depth profiles (Fig. 11b), the intensities of the Li^−^, F^−^, and P^−^ signals exhibit continuous increase from the surface (0.15 M, in contact with the cathode) to the bulk of gel (1.5 M). It has been shown that LiPF_6_ is unable to initiate the gelation of electrolyte at a low concentration of 0.15 M (Fig. 11c). Therefore, the gradient configuration of the IsIP electrolyte is formed with reduced solidification degree from the separator side to the cathode side. The resulting gel polymer served as a chemical barrier against the shuttle effect. Moreover, it has been demonstrated that in situ formed polymer electrolytes can indeed improve the uniform deposition of Li owing to the inherent elasticity of the polymer [82]. This design presents a promising solution to address both the physical and chemical interface challenges encountered in solid-state batteries.

Liu et al*.* [85] utilized pentaerythritol tetraacrylate (PETEA) featuring high ionic conductivity in the process of in situ gelation. The precursors containing 1.5 wt% PETEA (monomer) and 0.1 wt% azodiisobutyronitrile (AIBN, initiator) was dissolved in 1 M LiTFSI/DOL/DME with 1 wt% LiNO_3_, and subsequently injected into the commercial separator and filled into the battery. The assembled battery was aged for 2 h, then heated in a vacuum oven at 70 °C for 2 h to ensure thorough polymerization of PETEA. An integrated structure with close interfacial contact was developed between the GPE and the bare sulfur cathode. High-strength PETEA-based GPE pre-covered the cathode surface, inducing the formation of a flexible protective layer that enables the sulfur cathode to retain its structural integrity and stability despite the volumetric change of sulfur particles during the charge/discharge process. The protective layer is capable of effectively separating the sulfur electrode from the organic electrolyte, and thus suppressing the continuous interfacial reaction and polysulfide dissolution (Fig. 11d). Given the potential issues arising from uneven polymerization and the limited mechanical strength of the in situ electrolyte can lead to local short circuits, the aforementioned batteries still contain polyolefin separators as a barrier layer to avoid local short circuits. In order to further enhance the mechanical strength of GPE to eliminate commercial separators and to optimize ion channels in porous media to prevent polysulfide diffusion, this group developed an acrylate-based hierarchical electrolyte (AHE) for Li–S batteries [86]. The AHE was fabricated through the process of in situ gelation of PETEA-based GPE by AIBN initiator within polymethyl methacrylate (PMMA)-based electrospun fiber network. The PETEA-based GPE with jelly-like consistency enwrapped the PMMA-based electrospun fiber and sufficiently filled the overlap pores, contributing to excellent strength and high ionic conductivity of 1.02 × 10^−3^ S cm^−1^. As a result, the electrochemical performance of Li–S batteries was significantly improved, showing 91.9% capacity retention after 500 cycles at 3C. Free radical polymerization reactions initiated by AIBN have the advantage of being easy to control. However, it should be noted that AIBN initiator exhibits poor compatibility with lithium metal anodes. The chemical or electrochemical stability between monomers or initiators and electrodes is primary concern for developing advanced solid-state batteries through the in situ polymerization methods. Therefore, it is necessary to regulate the in situ polymerization process and explore novel initiators to develop SSEs with excellent physicochemical and electrochemical characteristics.

The components of precursors and initiators play a crucial role in the polymerization process because their functions determine the properties of the polymer electrolytes. In addition, due to the high activity of lithium anode, it is essential to optimize the polymerization conditions to build solid-state lithium metal batteries with excellent electrochemical properties. Generally, the monomers are mainly categorized into organic compounds containing unsaturated C=C bonds, cyclic carbonates, cyclic esters and cyclic ethers [112]. Among them, the ether-based monomers (e.g., DOL) have the best chemical/electrochemical stability against lithium metal anodes and their polymerization reaction conditions are relatively mild, making them ideal for application in solid-state Li–S batteries. Compared with traditional ex situ polymerization methods, in situ polymerization not only simplifies the preparation process but also forms an integrated structure that enhances the interface compatibility between the electrode and electrolyte. Furthermore, it effectively mitigates the notorious “shuttle effect” and increases the cycle life of solid-state Li–S batteries. Despite this, there are a few obstacles to overcome while using this strategy. For example, there may be flammable liquid electrolytes in this design, thereby posing safety hazards in the battery operation. Additionally, the inevitable parasitic reactions of the polymerization process, such as side reaction between the lithium metal and the initiator, can negatively affect the electrochemical performance of the solid-state batteries. During the charge/discharge process, these residual monomers probably undergo decomposition and accumulate on the electrode surface, resulting an increase in the interfacial resistance and degradation of cycle performance. Therefore, the selection of suitable polymerization conditions and the minimization of residual monomer content are of utmost significance. Further systematic investigations into various influencing factors, such as initiator activity, amount of initiator, and polymerization temperature, should be intensively progressed so as to advance in situ polymerization of solid-state lithium batteries [119].

## Pouch Cells Toward Practical Solid-State Li–S Batteries

Based on the distinctive characteristics of Li–S chemistry, various effective strategies have been proposed to improve the performance of solid-state Li–S batteries [8, 20, 23, 120, 121]. Although remarkable advancements have been observed in solid-state Li–S batteries at the laboratory level, such as achieving high specific discharge capacity of 980 mAh g^–1^ after 2000 cycles [61], most of the excellent performances are achieved with low sulfur loading (around 1 mg cm^–2^), thick electrolyte (> 500 µm), and excessive lithium anode (typically 100 times in excess for a 1.0 mAh cm^–2^ cathode). It is crucial to acknowledge that such parameters lead to a substantial sacrifice in the overall energy density. Furthermore, in the typical coin cells used in laboratories, the active areas of the cathode and anode are relatively small (usually smaller than 3 cm^2^). By contrast, pouch cells containing larger active areas often leading to more nonuniform current densities and shorter lifespans. In the pursuit of commercial level of energy storage devices, the scaling up of the SSEs preparation, interfacial strategies, and battery assembly is a non-negligible part in the ongoing research of solid-state batteries.

### Pouch Cells with Inorganic SSEs

In conventional liquid systems, the internal components of a pouch cell are configured in folded units, including cathodes, anodes, a separator, current collectors, and aluminum-plastic film package [121]. The cell configuration and assembly procedure of pouch cells vary significantly as a consequence of the difference of SSEs. For inorganic SSEs, the inherent rigidity renders them incapable of folding and rolling like a flexible polymer separator. Among the aforementioned SSEs, sulfide SSEs are the most popular SSEs employed in solid-state Li–S batteries [122, 123]. When compared to other types of inorganic SSEs, sulfides exhibit higher ionic conductivity, favorable processability, and excellent compatibility with sulfur cathodes. Hence, it is widely acknowledged that sulfide-based Li–S batteries hold great promise as all-solid-state batteries for real applications. In a typical process for solid battery assembly, cathodic composites containing sulfur or Li_2_S) are mixed with sulfide SSE powders, and then pressed into a compacted pellet under external pressure. The cathode pellet is then integrated with SSE pellet and Li anode to further form a sandwich-structured cell configuration.

**Fig. 12:**
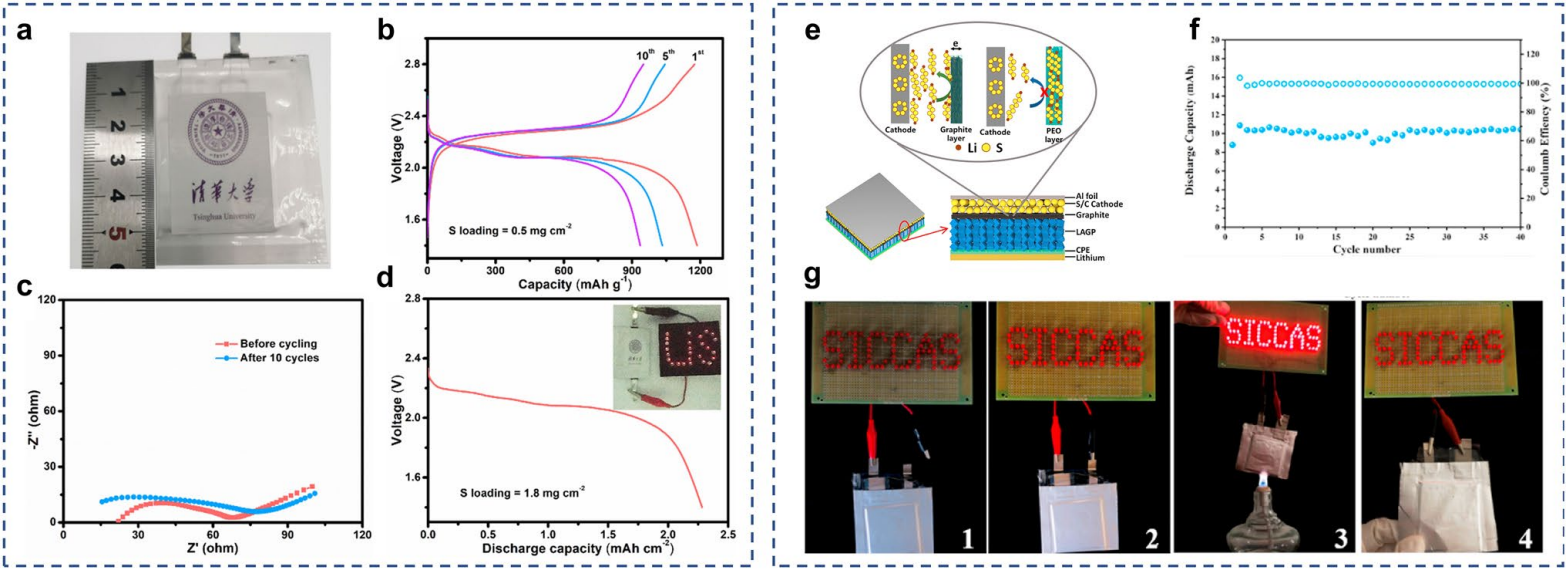
**a** Optical photograph and **b** the charge–discharge voltage profiles of solid-state Li–S pouch cells at 0.01 C, and **c** their EIS profiles before and after cycling. **d** Discharge profile of the pouch cell with 1.8 mg cm^−2^ sulfur loading, and the inset is the optical photograph of illumined LEDs by pouch cell. **a–d** Reprints from Ref. [124] with permission. Copyright 2020, Wiley–VCH Verlag GmbH & Co. KGaA, Weinheim. **e** Schematic illustration of the solid-state Li–S batteries with the LAGP ceramic modified by graphite and CPE. **f** Cycling performance of the soft package Li–S cell based modified LAGP ceramic at 60 °C. **g** Li–S cell lights up the “SICCAS” LED lamps at (1) open circuit, (2) connected circuit, (3) burning, (4) after fractured. (**e–g**) Reprints from Ref. [70] with permission. Copyright 2019, Elsevier B.V

Yuan et al*.* [124] fabricated all-solid-state Li–S pouch cells with dimension of 3 × 3 cm^2^ to investigate the practical feasibility of sulfide SSEs (Fig. 12a). Under external pressure, sulfur electrode sheet (3 × 3 cm^2^), Li_6_PS_5_Cl electrolyte sheet (3.3 × 3.3 cm^2^, 380 µm in thickness), and lithium metal anode with a thickness of 100 μm were stacked in a sandwich-type structure. The pouch cell with 0.5 mg cm^–2^ sulfur loading exhibited a high discharge capacity of 1169 mAh g^–1^ at 0.01C at 60 °C (Fig. 12b). After 10 cycles, a reversible capacity of up to 950 mAh g^−1^ was preserved. Furthermore, the interface impedances of the pouch cell remained below 70 Ω in the initial state and after 10 cycles, demonstrating the interfacial stability (Fig. 12c). To meet the need for high-energy density, the mass loading of sulfur cathode was increased to 1.8 mg cm^–2^, resulting in an areal capacity of more than 2.3 mAh cm^–2^ (Fig. 12d). This remarkable achievement holds significant promise for its practical application. In the research of solid-state soft-packed Li–S batteries by Hu et al*.* [125], the following procedure was involved: Firstly, the composite sulfur cathode (S/C/Li_6_PS_5_Cl, 2 × 3 cm^2^) and Li_6_PS_5_Cl SSE sheet (2.5 × 3.5 cm^2^) were compressed under cold isostatic press (under a pressure of 360 MPa). Subsequently, a lithium foil (2.2 × 3.2 cm^2^) was pasted on the opposite side of the SSE sheet. After that, the lugs were pre-welded on the current collector via an ultrasonic welding machine, and the cell was eventually assembled into aluminum-plastic film package. The Li–S pouch cell delivered a high discharge capacity of 9.2 mAh. Even under a harsh test situation involving fragmentation, the pouch cell was able to light up the light-emitting diode (LED) lamps, validating the superior safety of SSEs as compared to the liquid electrolyte systems.

As discussed in the Sect. 2.4, the interfacial contact between the rigid inorganic electrolyte and the solid electrodes is consistently poor, which impedes the electrochemical performance of solid-state batteries. The interfacial modification by employing flexible polymer interlayers/coatings can greatly improve the area contact and ionic conduction between the inorganic SSEs and the electrodes [126, 127]. To improve the interfacial contact between LATP SSE and Li anode, Li et al*.* [110] introduced a composite polymer electrolyte (CPE) layer with a thickness of 200 µm on the LATP SSE (Fig. 12e). In addition, an electrically conductive graphite layer (2 µm) was applied at the SSE/S interface to improve the utilization of sulfur. Consequently, the soft-packed battery delivered stable capacities of exceeding 10 mAh for 40 cycles (Fig. 12f). Moreover, the pouch batteries could light up the LED lamps even under harsh circumstances (including fire and rupture) (Fig. 12g), highlighting the promising application prospects in terms of high-energy density and exceptional safety.

**Fig. 13:**
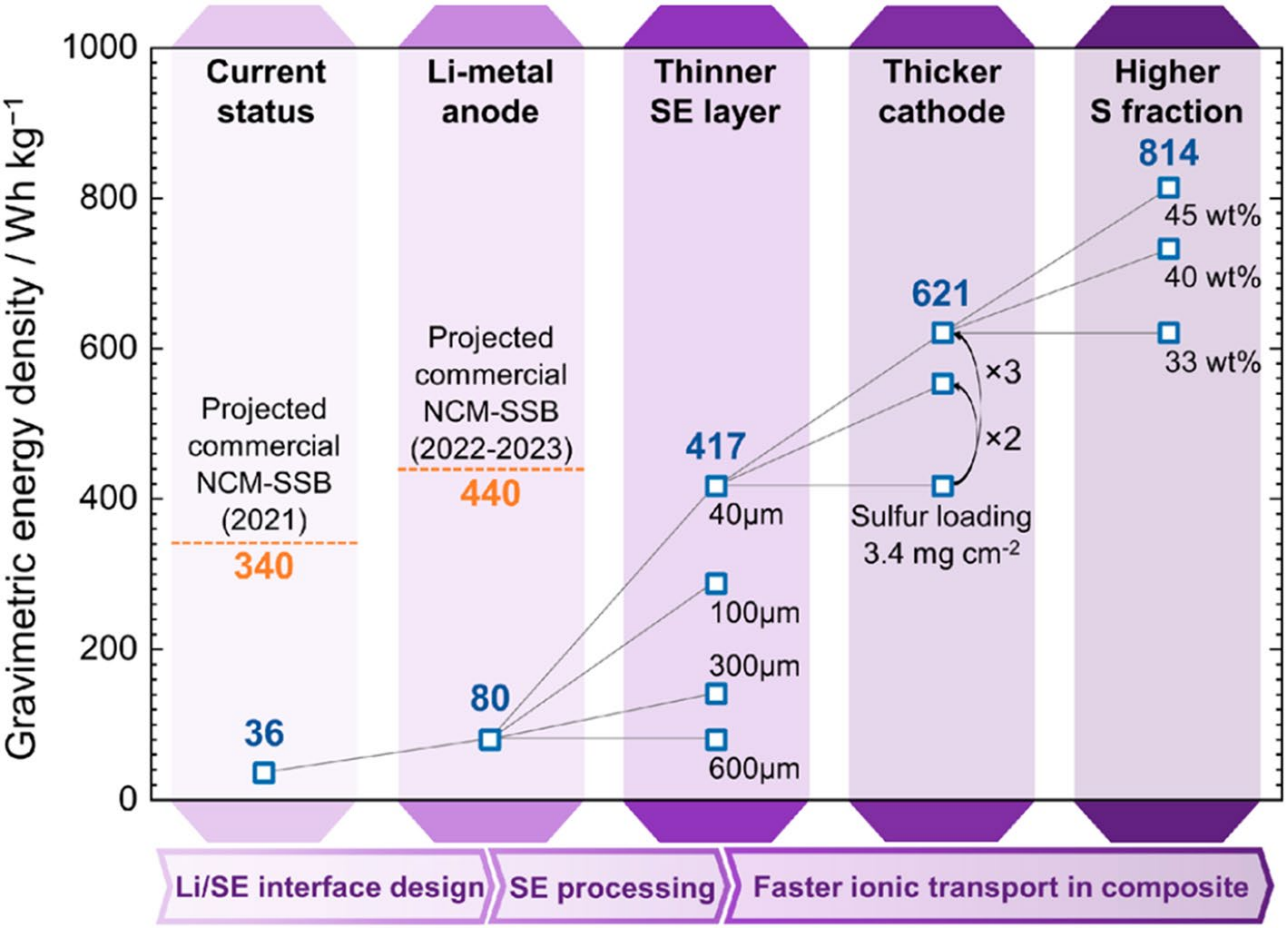
Roadmap toward the practical solid-state Li–S batteries according to the currently reported cell performance. Reprint from Ref. [128] with permission. Copyright 2021, American Chemical Society

However, the inorganic SSEs used in most of the developed solid-state Li–S batteries exhibits a thickness of up to 700 µm. As a result, the state-of-the-art solid-state Li–S batteries, despite with high sulfur loading, have low overall energy densities. According to the calculation conducted by Yang et al*.* [123], to achieve high-energy density of 500 Wh kg^–1^, the thickness of the electrolyte pellet should be less than 200 μm. As supposed in Fig. 13, the employment of a lithium metal anode, a thin SSE, and high sulfur loading will significantly enhance the solid-state battery performance, reaching the gravimetric energy density above 500 Wh kg^–1^ [128]. Therefore, it is necessary to optimize the thickness of the SSEs in order to fulfill the total package weight criterion and reduce the in-plane resistance of the practical system. Furthermore, the currently employed fragile pellet structures, mostly prepared by mechanical pressing, can only support very small battery sizes. In a brief summary, the development of inorganic SSE-based Li–S batteries is still its infancy. Flexible, thin, and low-cost electrolyte films with exceptional mechanical robustness and toughness are ideal structures for the future SSEs. In this context, upgrading of thin film deposition methods or the solution-processed techniques may contribute to the manufacturing and assembly of practical solid-state batteries.

### Pouch Cells with Polymer SSEs

The configuration and assembly of Li–S batteries that use polymer SSEs, including solid polymer electrolyte (SPE), gel polymer electrolyte (GPE), and composite polymer electrolyte (CPE), are very similar to those of liquid systems (layer-by-layer structure), where the liquid electrolyte and separator are replaced by flexible polymer electrolyte membrane. The most attractive advantage of polymer-based Li–S batteries over liquid electrolyte is the improved safety by replacing or confining the flammable liquid electrolyte. Therefore, polymer-based solid-state batteries can be successfully implemented at mass production by following a similar protocol to those used in the manufacturing of conventional lithium-ion battery [123].

**Fig. 14:**
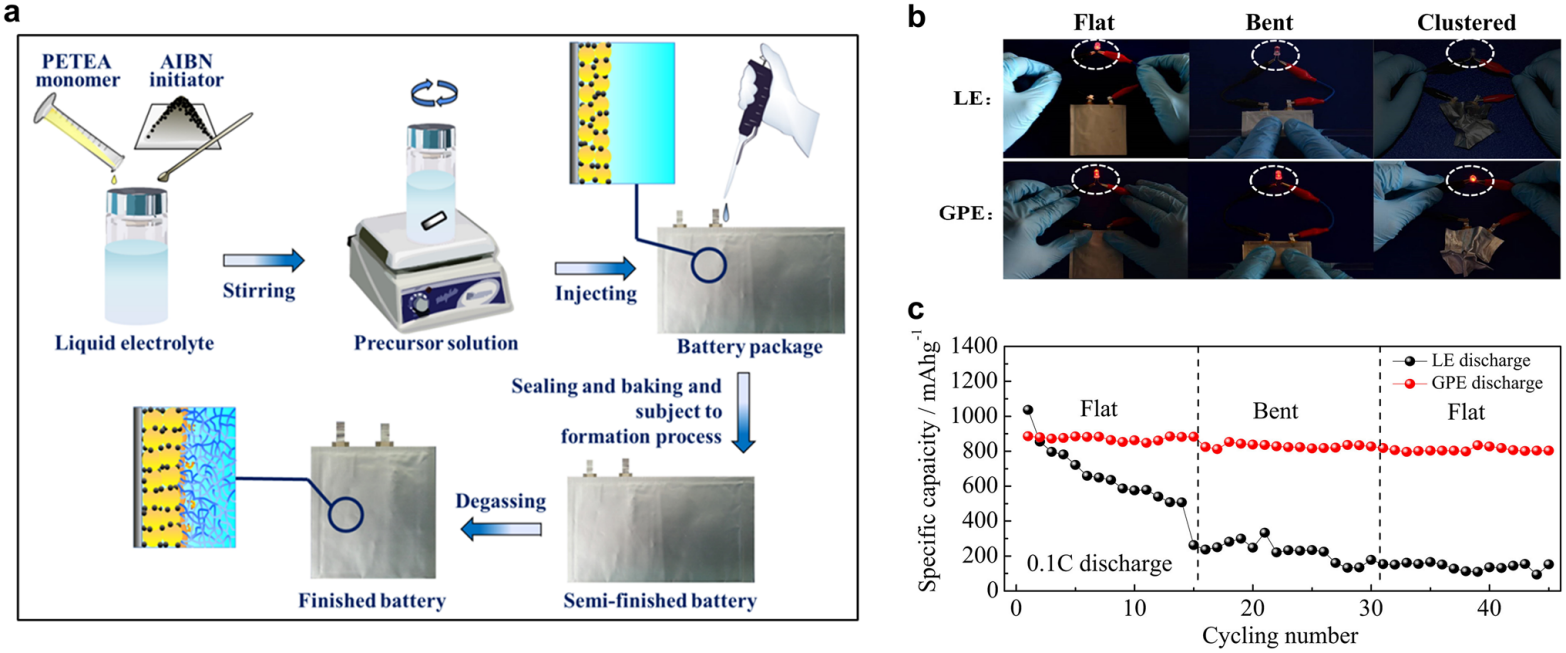
**a** Stepwise procedure of the in situ assembly of Li–S batteries. **b** Digital images of LEDs powered by Li–S batteries based on a liquid electrolyte (LE, upper panels; S/LE/Li cell) or PETEA-based GPE (lower panels; S/GPE/Li cell) under various deformed states (i.e., flat, bent and clustered states). **c** Cycling performances of S/LE/Li and S/GPE/Li cells under flat and bent states at 0.1C. Reprinted from Ref. [85] with permission. Copyright 2016, Elsevier Ltd

Among the polymer SSEs, GPE with high ionic conductivity and outstanding interfacial contact demonstrates unprecedented adaptability to the packaging technologies used in the current battery systems. Hence, its large-scale preparation and subsequent application in actual industrializations is fully foreseeable. Liu et al*.* [85] fabricated a soft-packed quasi-solid Li–S battery via in situ polymerization of electrolyte, wherein sulfur served as the cathode, PETEA-based GPE functioned as the electrolyte, and lithium strip as the anode. The assembly procedure of the pouch cell, as depicted in Fig. 14a, bear a striking resemblance to that employed in liquid systems. First, the Al and Ni strips were joined anchored to the side of cathode and anode as the electrode lugs, respectively. Then, the electrodes and separator were laminated together to form the battery core and assembled into aluminum-plastic film packages. The next step involved injecting the precursor solution of PETEA into these packages and sealing batteries under vacuum. Subsequently, the assembled cells were aged at room temperature for 6 h to ensure the thorough wetting of the electrodes, and then subjected to polymerization process at 70 °C under 0.25 MPa. Finally, the pouch batteries were aged at 25 °C for 12 h followed by a degassing process. The pouch battery retained discharge capacity of 803 mAh g^–1^ with impressive capacity retention of 91.78% after 45 cycles, and exhibited excellent cycling stability under both flat and bent states (Fig. 14b, c). Liu et al*.* [84] fabricated soft-packed quasi-solid Li–S batteries by employing the in situ cationic polymerization of ether-based liquid electrolyte (containing DME and DOL) under ambient temperature. During the assembly of pouch cell, the precursor solution was injected into the separator as in the liquid systems. Subsequently, the assembled batteries were left to stand for a period of time to form GPE in the presence of LiPF_6_. The resultant soft-packed S|GPE|Li battery could power LED lamps under mechanical deformations such as bending or folding. The GPE displayed a notable decrease in mobility along with higher mechanical strength, which contributed to maintaining stronger and more stable interface attachment, and consequently, the battery demonstrated improved adaptation to shape transformation. Despite the advantages and progresses in the development of polymer-based solid-state batteries, the challenges of sulfur loading, areal capacity ratio of negative-to-positive electrode (N/P ratio), and gravimetric energy density are still unsatisfactory and unsuitable for commercial application.

### Challenges of Interfacial Strategies Toward Practical Li–S Batteries

For inorganic SSEs, the most popular method for enhancing the interfacial contact is to apply external pressure. Unlike flexible polymer separators, inorganic SSEs cannot be folded and wound due to their inherent rigidity and brittleness. During the battery assembly, the cathode pellet is integrated with SSE pellet and Li anode to form a sandwich-structured cell configuration under high pressure. In order to obtain better contact, operation stack pressure is also provided during the cell test. However, the battery performance (e.g., energy density and rate performance) of these solid-state batteries is far from reaching the requirements for commercial applications. The primary obstacles include high interfacial impedance, sluggish charge-carrier transport, and interfacial degradation associated with volume changes and electrolyte decomposition [23]. Under molten lithium treatment and heating pretreatment conditions, significant enhancement of the interfacial contact between the Li anode and the inorganic SSE can be achieved. However, it is important to consider both safety and cost factors when evaluating the industrial application of these techniques. In addition, the potential exacerbation of interfacial parasitic reactions between the molten lithium and the electrolyte is another concern.

On the other hand, the existing synthetic techniques for inorganic SSE pellets are constrained by operation pressure and limited sizes. In order to fulfill the total package weight criterion and reduce the in-plane resistance of the practical system, it is necessary to optimize the thickness of the SSEs [129]. Flexible, thin, and low-cost electrolyte films with exceptional mechanical robustness and toughness are ideal structures for the future SSEs. In this context, upgrading of the thin film deposition methods or the solution-processed techniques may contribute to the manufacturing of practical solid-state batteries. The introduction of a conformal interfacial buffer layer by vapor deposition or solution casting methods has shown notable superiority in improving the interfacial contact and exhibit promising prospects for industrialization. For example, the preparation of silicon coatings using CVD is a well-established technology in the semiconductor industry and is expected to be directly applicable to the mass production of solid-state lithium batteries.

Li–S batteries using polymer SSEs are configured and assembled in a process that is very similar to those of liquid systems. Especially in the case of the in situ polymerization, the polyolefin separator serves as the electrolyte scaffold to support the solidification of GPEs. Therefore, the mass production of polymer-based solid-state batteries can be successfully achieved by following similar protocols used in the manufacturing of conventional lithium-ion batteries. In terms of interfacial resistance, polymer SSEs are in favorable contact with electrodes owing to their inherent softness and toughness. The most prominent challenge for the implementation of polymer SSEs in practical Li–S batteries lies in two aspects [123]: (1) the significant difficulty in achieving both high room-temperature ionic conductivity and superior mechanical properties simultaneously; (2) the shuttle effect resulting from the solid–liquid phase reaction of sulfur. In a brief summary, the development of solid-state Li–S batteries is still its infancy. Multiple scientific, technological and manufacturing issues with solid-state Li–S batteries still need to be addressed before meeting the requirements for practical applications.

## Conclusion and Perspective

The establishment of a favorable ionic conduction and conformal interface between the electrodes and the solid electrolytes is of paramount importance in solid-state batteries. Due to the distinctive redox mechanism and material characteristics inherent in sulfur cathodes, the interfacial challenges encountered in solid-state Li–S batteries are not exactly the same as those of Li-ion batteries. Recently, continuous progresses have been made in the interfacial design, with the aim of enhancing the physical contact between electrodes and SSEs. It is noteworthy that the physicochemical properties of the anode and cathode materials exhibit marked differences. For example, lithium anode is highly active to many reagents, while sulfur/carbon composite cathode is relatively inert. As a result, the methodologies employed to reduce the interfacial impedance of the anode/SSE and cathode/SSE exhibit dissimilarities. Generally, the processing sequence for S/SSE precedes that of Li/SSE. This is mostly due to the inherent high reactivity of metallic lithium, which readily reacts with many chemical reagents. In contrast, sulfur or Li_2_S exhibits comparatively inert nature and is less susceptible to environmental factors. The use of such processing sequence has been shown to efficiently mitigate the potential contamination or damage to the Li anode caused by chemical solvents, mechanical high pressure, and other relevant elements. In this review, we have provided an overview of the experimental methodologies to enhance the interfacial contact in the processes of interfacial design and battery assembly in solid-state Li–S batteries. To date, a variety of strategies have been employed to achieve enhancements, such as mechanical pressing, vapor deposition, molten lithium treatment, polymer modification, slurry casting, and in situ polymerization. Although significant progresses have been achieved in the research of tuning the optimal interfaces, further endeavors are requisite to promote the development of solid-state Li–S batteries, and there are some challenges to be solved:

1. The electrochemical performances of the state-of-the-art solid-state Li–S batteries are still inferior to those of liquid electrolyte systems, as indicated in Table 1. The electrolyte/electrode interfacial resistance appears to be the main limitation, leading to the large polarization and unsatisfactory rate capability. The next step toward achieving the high-energy density and high-power density Li–S batteries is the development of SSEs that allows fast Li-ion transport throughout sulfur cathodes (in particular with high sulfur loading), intimate interface between electrodes and electrolytes, and good interfacial stability with both sulfur cathodes and Li anodes.
2. Scaling up the interfacial technologies is still confronted with numerous obstacles as discussed at the end of each section. Currently, among the aforementioned interfacial strategies, the processes that are more compatible with the large-scale production toward battery manufacturing are mechanical pressing, slurry casting and in situ polymerization. However, there are still some issues that need to be addressed in these processes. For example, mechanical pressing is a solvent-free technique where the pre-step entails mixing the electrode material with electrolyte under high shear and/or high-pressure processing. Achieving the homogeneous and rapid mixing of these solid materials on a large scale is a challenge. Therefore, the development of low-cost and advanced assembling techniques for solid-state batteries (on par with conventional Li-ion batteries manufacturing costs) is expected to be critical for their commercial success.
3. To uncover the obscure fundamental mechanisms of solid-state Li–S batteries is important. SSEs serve dual functions as ion conductors and separators, necessitating their high ionic conductive but electrical insulated properties. Meanwhile, the electrodes demand high electronic/ionic conductivity to achieve excellent rate performance. It remains to be discussed whether the electrode/electrolyte interphases can fulfill their designated functions after the interfacial integration. The process of Li plating/stripping is a dynamic process, and the huge volume change of Li anodes will result in partial delamination and incompatible contact between the Li and SSEs. As a consequence, this can give rise to an unstable interface and the accumulation of local stress, further exacerbating the growth of dendrites. In addition, due to the unique sulfur redox chemistry, the interfacial theories of conventional Li-ion batteries mismatch with those of the Li–S batteries. The in-depth understanding and development of the critical mechanisms are necessary for elucidating the actual processes happening at the electrode/electrolyte interfaces/interphases during charge/discharge cycling of Li–S batteries. For example, the application of different interfacial methods on the electrode materials has dramatic multiple consequences on the electrochemical processes.
4. The properties and behaviors of the electrode/electrolyte interface are critical for achieving solid-state batteries with high-energy density and stable cycling life. To understand the dynamic evolution processes of the batteries, comprehensive and in-depth examination of the microstructure, phase composition, and chemical environment of the interfaces using multiple characterization techniques is required. In the assembly of solid-state batteries, mechanical pressing, liquid casting, and vapor deposition methods are usually employed to obtain enhanced electrode/electrolyte interfacial contact. Unlike batteries using liquid electrolytes, the tightly embedded and fused interfaces cannot be fully exposed, making it difficult to acquire insights into their interfacial reactions and kinetics. As a result, it is important to consider the design of characterization approaches when assessing the interfaces of solid-state batteries. Advanced characterization techniques are suggested to be used for the precise investigation of the interfacial processes in solid-state Li–S batteries, such as in situ X-ray diffraction, in situ/ex situ solid-state nuclear magnetic resonance (NMR), in situ Raman spectroscopy, X-ray absorption spectroscopy (XAS), time-of-flight secondary-ion mass spectrometry (TOF–SIMS), and in situ electrochemical techniques. Current understanding on the underlying mechanisms of electrode/electrolyte interface is still very limited due to the considerably complicated reaction process and technological limitation. Therefore, it is necessary to characterize and elucidate ion transport mechanism and interfacial evolution of SSEs from a microscopic perspective by using these advanced characterization techniques.
